# Equity impact and cost-effectiveness of a community health worker breast cancer educational programme in rural South Africa: a modelling study

**DOI:** 10.1136/bmjopen-2025-114908

**Published:** 2026-04-21

**Authors:** Esther Z Chanakira, Chloe Thomas, Jacqueline Miot, Olena Mandrik

**Affiliations:** 1 Sheffield Centre for Health and Related Research, School of Medicine and Population Health, The University of Sheffield, Sheffield, UK; 2 Health Economics and Epidemiology Research Office, University of Witswatersrand, Faculty of Health Sciences, Johannesburg, South Africa

**Keywords:** PUBLIC HEALTH, Health Equity, HEALTH ECONOMICS, Africa South of the Sahara, Early Detection of Cancer

## Abstract

**Abstract:**

**Introduction:**

Breast cancer is a leading cause of cancer-related death among women. Women with lower income, those living in rural areas and women of Black ethnicity are more likely to be diagnosed at advanced stages and have poorer survival outcomes. Reducing these inequities is an important public health priority. This study aimed to identify a cost-effective strategy for reducing breast cancer-related inequities and to evaluate the equity impact of the intervention across population subgroups.

**Methods:**

We developed a novel individual-level microsimulation model to assess both the equity impact and cost-effectiveness of a community health worker-led education intervention in rural areas. The model, with annual cycles, simulated rural and urban breast cancer populations in South Africa using data from national and regional cancer datasets and followed individuals over a lifetime horizon. Costs were estimated from the provider perspective and outcomes included life-years, quality-adjusted life-years (QALYs), and incremental cost-effectiveness ratios (ICERs) compared with three willingness-to-pay thresholds (ZAR 58 018/ZAR 109 468/ZAR 328 408). Parameter uncertainty was explored using probabilistic sensitivity analysis. Equity impact was evaluated by estimating changes in age-standardised all-cause mortality across subgroups defined by place of residence (rural vs urban) and ethnicity (Black vs non-Black), using both absolute (rate differences) and relative (rate ratios) measures.

**Results:**

The intervention generated average gains of 0.35 life-years and 0.31 QALYs per patient across the breast cancer population. Inequities by residence decreased, with an absolute reduction of 229.65 per 1000 patients with breast cancer in the age-standardised mortality rate difference, and a relative reduction in the rate ratio of 0.80. By ethnicity, absolute and relative reductions of 110.26 per 1000 patients and 0.27, respectively, were observed between Black and non-Black populations. The intervention was cost-effective, with an ICER of ZAR 44 124 (I$6036) per QALY gained, which is below all three willingness-to-pay thresholds considered.

**Conclusions:**

Community health worker programmes represent a cost-effective strategy to reduce breast cancer-related inequities. Their integration into national cancer control plans in low-income and middle-income countries should be prioritised and supported.

**STRENGTHS AND LIMITATIONS OF THIS STUDY**

- This work addresses a major evidence gap, delivering the first cost-effectiveness evaluation and equity impact analysis on early detection of breast cancer in a low-income and middle-income country setting.
- This analysis is underpinned by a proven community health worker model, drawing on the well-documented success of community health worker-led programmes for diabetes, hypertension and HIV/AIDS in South Africa, all of which are cost-effective and locally accepted.
- By incorporating both a South African specific willingness-to-pay threshold and the international WHO-CHOICE benchmark, the study delivers a more comprehensive and contextually grounded assessment of cost-effectiveness while also enabling international comparability of study findings.
- The intervention effect used in this study is drawn from a randomised controlled trial conducted in Rwanda, which may limit the applicability of the findings to South Africa, which was explored during sensitivity analysis.

## Introduction

 Breast cancer is the most diagnosed cancer among South African women. The National Cancer Registry reported an age-standardised incidence rate of 33.95 cases per 1 00 000 females in 2019, while GLOBOCAN estimated it to be 47.8 cases per 100 000 in 2022.^1 2^ Late-stage diagnoses remain a major challenge, with 43% of patients presenting at stage 3 and 20% at stage 4.^3^ Earlier diagnosis is strongly associated with improved survival, with a study in Johannesburg concluding that patients diagnosed at stage 1 and 2 had significantly better 3-year survival compared with those at stage 3 or 4 (HR: 2.8, 95% CI 1.9 to 4.1).^4^

Delays in diagnosis are often driven by low symptom awareness and fear of treatment options such as mastectomy and chemotherapy.^5^,^7^ These delays disproportionately affect Black women, and those with lower education and socioeconomic status, many of whom reside in rural areas due to apartheid-era disparities.^8^ The South African Breast Cancer and HIV Outcomes study found that women diagnosed at a tertiary facility serving a predominantly rural population were approximately three times more likely (OR: 2.89, 95% CI 1.40 to 5.97) to receive a late-stage breast cancer diagnosis compared with those diagnosed at a facility primarily serving an urban population.^9^

South Africa currently lacks a national breast cancer screening programme. Community health workers (CHWs), who are embedded in primary healthcare centres (PHCs) and share cultural and linguistic ties with the communities they serve, represent a promising platform for improving early detection. CHWs already play an important role in chronic disease management and have demonstrated cost-effectiveness in conditions such as HIV and diabetes.^10 11^ However, they are not currently trained to recognise cancer symptoms.^12^

A review of interventions for early detection of breast cancer in low-income and middle-income countries (LMICs) indicated that CHW-delivered programmes can improve symptom awareness resulting in early detection.^13^ A cluster randomised controlled trial conducted in Rwanda demonstrated that a CHW educational intervention significantly improved early-stage breast cancer presentation. In the intervention arm, the proportion of early-stage cases increased by 27.4% compared with the control arm.^14^ The trial evaluated a comprehensive approach in which CHWs were trained to recognise breast cancer symptoms and encourage early presentation, while PHC nurses received training in symptom recognition, clinical breast examinations and the use of clinical algorithms. Despite such promise, no prior evidence exists on the cost-effectiveness of CHW-led interventions for breast cancer in LMICs. This study therefore aims to evaluate the cost-effectiveness and equity impact based on place of residence and ethnicity of a CHW educational intervention for early breast cancer detection in rural South Africa, compared with current care.

## Methods

A new microsimulation model was developed using R language to allow for detailed representation of individual-level heterogeneity in costs, disease outcomes and intervention impact.

### Model population

Baseline model population included women aged 18–99 with a first-time breast cancer diagnosis. A synthetic population was generated using iterative proportional fitting with data from the South African Breast Cancer and HIV Outcomes study,^9^ a prospective cohort study of newly diagnosed patients across multiple hospital sites. Weights were calibrated to national incidence distributions from the South African National Cancer Registry (2019),^1^ by age and race. To adjust for the study’s urban bias, rural–urban proportions (30.56% vs 69.44%) from the 2016 Demographic and Health Survey were incorporated as an additional constraint in the iterative proportional fitting process.^15^

### Model structure

**Figure 1:**
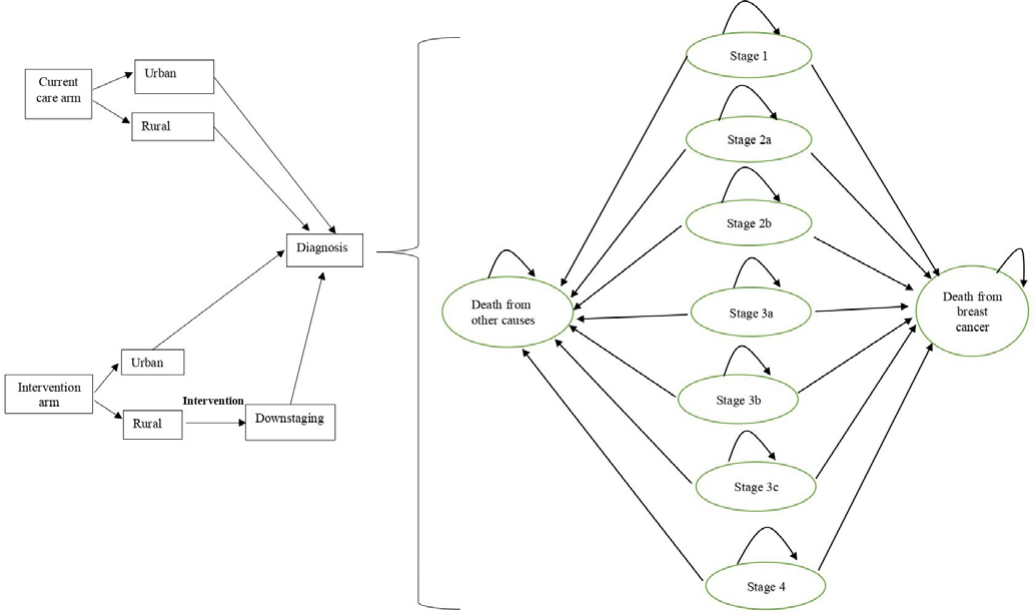
Model structure diagram showing model arms and the Markov model health states for survival postdiagnosis.

The model consists of nine mutually exclusive health states based on the numeric staging system: breast cancer stages 1, 2a, 2b, 3a, 3b, 3c and 4, as well as death from breast cancer and death from other causes.^16^ Individuals enter the model in their stage at diagnosis. Rather than simulating disease progression, the model estimates survival by applying stage-specific mortality probabilities, with transitions occurring only to death from breast cancer or other causes. Transition probabilities are stratified by patient characteristics, including age, education level, time since diagnosis and place of residence. The model uses a 1-year cycle length and a 90-year lifetime horizon. Model structure is illustrated in figure 1.

### Model parameters

Full model parameter details, including sources, assumptions and uncertainty ranges, are provided in the supplementary methods, with a summary presented here.

#### Mortality data

Stage-specific all-cause survival was estimated from 3-year Kaplan-Meier curves obtained from the prospective African Breast Cancer-Disparities in Outcomes (ABC-DO) cohort study.^17^ Survival estimates were adjusted for place of residence and highest level of education to reflect observed differences in outcomes.^17^ These estimates were extrapolated to 10 years, after which an assumption of no further breast cancer deaths was applied. This assumption is supported by clinical evidence and epidemiological data, including long-term survival patterns reported by the Surveillance, Epidemiology and End Results programme in the USA, which demonstrates that breast cancer mortality risk significantly declines several years after diagnosis and approaches that of the general population beyond the 10-year mark.^18^ Deaths were then partitioned into death due to breast cancer and death from other causes using South African life tables and age-specific breast cancer mortality estimates from the Global Cancer Observatory (2022).^19 20^

#### Intervention effect

The intervention’s effect on stage at diagnosis was estimated using results from the trial conducted in Rwanda.^14^ Because the current stage distribution at diagnosis differs between Rwanda and South Africa, a relative effect approach was applied, resulting in estimated proportional reductions in late-stage diagnoses which were then applied to the South African rural population.^21^ Table 1 shows the resulting intervention effect.

**Table 1:** Stage proportions obtained from intervention study and resulting downstaging probabilities^14^

Stage at diagnosis	Proportion in trial control group	Proportion in trial intervention group	Difference (intervention–control)	Proportion expected to remain in same stage	Proportion expected to downstage	Probability of staying in same stage	Probability of downstaging to earlier stage
Early stage	0.222	0.500	0.278	0.222	0.000	1.000	0.000
Stage 3	0.444	0.278	−0.166	0.167	0.278	0.375	0.625
Stage 4	0.333	0.222	−0.111	0.222	0.111	0.667	0.333

#### Utility data

Due to the absence of South African-specific health-related quality of life (HRQoL) data, baseline HRQoL values were taken from a Colombian dataset, given Colombia’s similar upper middle-income status and income inequity.^21 22^ Utility multipliers reflecting quality of life reductions due to breast cancer and treatment were derived from a large Chinese study identified in a systematic review, which reported stage-specific utility values.^23^ After year 5, individuals were assumed to return to baseline utility, as disutility was considered to be primarily treatment related.^24^ Those with stage 4 disease retained reduced utility due to ongoing disease burden. For breast cancer deaths, a stage 4 relapse was assumed in the final year of life.

#### Costs

From the provider perspective, costs were estimated for diagnosis, staging, treatment and intervention implementation, expressed in 2023 South African Rand (ZAR) and International Dollar (I$) values, using a purchasing power parity (PPP) conversion rate of 7.31 ZAR per I$.^25^

Intervention costs included only components not currently part of routine public sector care, namely training of CHWs and mentorship of PHC nurses by advanced midwives.^14^ Given the limited proportion of CHW time expected to be devoted to breast cancer, associated operational costs were considered negligible. Training costs, adapted from a rural breastfeeding support programme and adjusted for inflation, were estimated at ZAR 2875 (I$393) per rural diagnosed case.^26^ Mentorship assumed the employment of 52 advanced midwives (one per district) to support an estimated 5336 rural cases in 2023, at an annual cost of ZAR 491 487 (I$67 235) per midwife, equating to ZAR 4790 (I$655) per case.^27^ The total mean intervention cost was therefore ZAR 7665 (I$1049) per rural diagnosed case.

Average diagnostic costs were applied at model initiation and reported separately for individuals under 35 and those 35 or older, reflecting differences in diagnostic procedures outlined in the National Breast Cancer Policy.^28^ Diagnostic costs were higher in the intervention arm due to additional investigations, with an average of 2.82 more patients presenting per breast cancer case in the trial (multiplier 3.82).^14^ Staging costs were ZAR 4908 (I$671) per confirmed case, while treatment costs ranged from ZAR 94 115 (I$12 875) for stage I to ZAR 110 689 (I$ 15 142) for stage IV. Recurrent disease was assumed to incur diagnostic costs and 57.8% of stage IV treatment costs in the final year of life, based on a study comparing first-time and recurrent diagnoses.^29^ A discount rate of 5% was applied to both costs and utilities in the base-case analysis, with additional sensitivity analyses conducted using alternative rates of 0%, 3% and 10%.^21^

### Cost-effectiveness analysis

Outcomes of interest were life-years saved (LYs), incremental quality-adjusted life-years (QALYs), incremental costs, incremental cost-effectiveness ratio (ICER) and net monetary benefit (NMB). Cost-effectiveness was determined using three willingness-to-pay (WTP) thresholds. The first threshold was obtained from a study conducted by Edoka and Stacey which measured the threshold for South Africa to be ≈53% of gross domestic product (GDP) per capita, considering 2022 GDP per capita which was ZAR 109 468 (I$14 975), a threshold of ZAR 58 018 (I$ 7937) per QALY was used.^30 31^ The WHO Choice thresholds of 1 and 3 times GDP per capita (ZAR 109 468 (I$14 975) and ZAR 328 408 (I$44 926)) per QALY were also used and will be referred to as WTP 2 and WTP 3, respectively.^32^ Probabilistic sensitivity analysis (PSA) was employed to explore the impact of uncertainty in model parameters on the outcomes of interest. Results from the PSA were presented as mean estimates with corresponding 95% credible intervals (CrI), representing the 2.5th and 97.5th percentiles of the simulated outcome distributions. These intervals reflect parameter uncertainty around the estimated outcomes for each strategy. Comparative assessments between strategies were based on incremental outcomes and cost-effectiveness measures derived from the probabilistic simulations. All model parameters included in the PSA, along with their assigned probability distributions, ranges and data sources, are summarised in online supplemental table S13; beta distributions were applied to proportions and probabilities, gamma distributions to cost parameters and lognormal distributions to utility values and relative risks.

### Equity analysis

The distribution of health benefits across subgroups under current care and the intervention was compared using age-standardised mortality rate (ASMR) from breast cancer and other causes over a 10-year horizon, as breast cancer mortality was only modelled within this period. Absolute and relative equity by place of residence and race were assessed respectively using rate differences $\begin{array}{l} (ASMR\;for\;disadvantaged\;population\;\\ \;-\;ASMR\;for\;advantaged\;population) \end{array}$ and rate ratios $\begin{array}{l} (ASMR\;in\;disadvantaged\;population\\ \;/\;ASMR\;in\;advantaged\;population) \end{array}$. Absolute changes in rate differences were calculated as the difference between intervention and current care arms, while relative differences were assessed through changes in rate ratios. The remaining proportion of relative inequity was estimated as $1-\frac{RRforinterventionarm-1}{RRforcurrentcarearm-1}$. Results were presented as the mean value and the associated 95% CrI.

### Scenario analysis

Scenario analyses were conducted to examine uncertainties beyond those addressed in PSA. A summary of these scenarios is presented here.

1. Lower intervention cost: Training costs for CHWs were annuitised over 5 years at 5% rate based on the assumption that skills obtained remained effective in that period. Diagnostic costs for individuals without breast cancer were also excluded.
2. Higher intervention cost: Added costs per rural diagnosed case included 1% of CHW time for breast cancer awareness in the community (ZAR 536/I$73),^33^ a weekly half-day breast clinic at PHCs (ZAR 17 516/I$2396) and follow-up visits for non-cancer patients (ZAR 457/I$63). The total cost per rural diagnosed case for this scenario was ZAR 26 174 (I$3581).
3. An alternative mortality data source based on a sub-Saharan African population-based registry dataset inclusive of the Eastern Cape registry in South Africa was used.^34^
4. An assumption was made that breast cancer mortality remained constant from year 3 to year 10.
5. A conservative intervention effect based on absolute difference, with a resulting probability of downstaging of 0.104 for stage 4 to 3 and 0.177 for stage 3 to early stage.
6. Higher intervention effect obtained by halving the probability of remaining in the same stage for stage 3 and 4.

Scenarios 3, 4, 5 and 6 were examined in the equity analysis due to their impact on ASMR.

### Patient and public involvement

Patients and the public were not directly involved as formal study participants. However, perspectives relevant to patients and affected communities were incorporated through informal engagement with an advocacy group representative, who reflected patient and community priorities related to breast cancer inequalities in South Africa. This engagement occurred during the early phases of the study through in-person stakeholder sessions, during which input from the advocacy representative contributed to shaping the overall research question. Their insights ensured that the study focus, particularly regarding the social determinants of breast cancer inequalities, aligned with the priorities, experiences and needs of affected communities.

The advocacy representative also contributed indirectly to the study design by informing key contextual elements incorporated into the study plan and conceptual model, including service delivery pathways, barriers to accessing care and practical constraints within the South African health system. This feedback played an important role in refining the model structure and assumptions to better reflect real-world conditions and the lived experiences of those impacted by breast cancer.

## Results

### Cost-effectiveness analysis

The intervention was estimated to result in 0.350 additional LYs and 0.305 additional QALYs per woman diagnosed with breast cancer. The average lifetime cost per person was higher in the intervention arm compared with the current care arm, with a difference of ZAR 13 428 (I$1837). An average ICER value of ZAR 44 124 (I$6036) was obtained from the model. Table 2 presents a breakdown of the costs and effects associated with the intervention and current care for the simulated breast cancer population.

**Table 2:** Model outcomes per person for the overall breast cancer population by model arm

	Life-years saved	QALYs	Costs (ZAR)	ICER (ZAR/QALY)
Current care arm	6.206 (6.015; 6.409)	5.924 (5.763; 6.096)	155 724 (153 546; 158 042)	
Intervention arm	6.557 (6.344; 6.776)	6.229 (6.049; 6.415)	169 152 (167 083;171 388)	
Incremental values	0.350 (0.312; 0.385)	0.305 (0.273; 0.336)	13 428 (12 996;13 854)	**44 124**

ICER, incremental cost-effectiveness ratio; QALYs, quality-adjusted life-years; ZAR, South African Rand.

**Figure 2:**
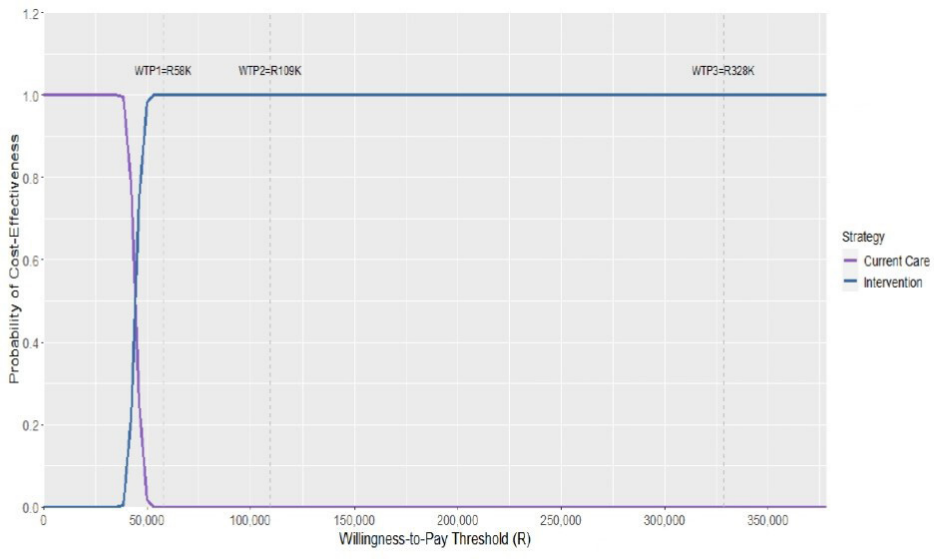
Cost-effectiveness acceptability curve showing the probability that the intervention is cost-effective across different willingness-to-pay (WTP) thresholds.

The intervention was cost-effective at all 3 WTP thresholds, with a probability of cost-effectiveness equal to 1.00 across the board (table 3). The cost-effectiveness acceptability curve depicts the probability that the intervention is cost-effective compared with current care in a range inclusive of the maximum and minimum WTP threshold values (WTP1 and WTP3) (figure 2). Online supplemental table S14 presents the cost-effectiveness outcomes for the additional discount rate scenarios of 0%, 3% and 10%.

**Table 3:** Cost-effectiveness results across WTP thresholds, reported as mean with 95% CrI

WTP (ZAR)	WTP (I$)		NMB (ZAR)	NMB (I$)	Probability of cost-effectiveness	Interpretation
58 018	8717	Current care arm	187 947 (177 769; 198 560)	25 711 (24 319; 27 163)	**1.00**	**Cost-effective**
		Intervention arm	192 228 (180 765; 203 799)	26 297 (24 728; 27 879)		
		Incremental values	4281 (2341; 6154)	586 (320: 842)		
109 468	16 447	Current care arm	492 711 (474 594; 512 278)	67 402 (64 924; 70 079)	**1.00**	**Cost-effective**
		Intervention arm	512 697 (492 312; 533 523)	70 136 (67 348; 72 985)		
		Incremental values	19 985 (16 414; 23 354)	2734 (2245; 3195)		
328 408	49 342	Current care arm	1 789 612 (1 737 225;1 846 393)	244 817 (237 651; 252 585)	**1.00**	**Cost-effective**
		Intervention arm	1 876 426 (1 817 792;1 937 631)	256 693 (248 672; 265 066)		
		Incremental values	86 814 (76 167;96 919)	11 876 (10 420; 13 258)		

CrI, credible interval; I$, International Dollar; NMB, net monetary benefit; WTP, willingness-to-pay; ZAR, South African Rand.

### Equity analysis

Within the first 10 years, ASMR was 714.81 per 1000 women diagnosed with breast cancer in the intervention arm vs 771.05 per 1000 patients in the current care arm. This overall reduction was driven by marked improvements in stage distribution. Among rural patients, the proportion diagnosed at an early stage increased substantially from 40% to 66% in comparison to 53% early-stage cases in the urban population in both model arms. Similarly, among Black patients, early-stage diagnoses rose from 37% to 65%. More modest gains were observed among non-Black patients, with early-stage diagnoses increasing from 58% to 61%. These stage shifts resulted in reduced inequity in ASMR across both axes; for rural vs urban populations, the rate difference fell from 280.38 per 1000 patients to 50.72 per 1000 patients and the rate ratio from 1.39 to 1.08. For Black versus non-Black, the rate difference declined from 381.96 per 1000 patients to 271.70 per 1000 patients and the rate ratio from 1.65 to 1.47. Table 4 presents the rate ratios and rate differences associated with each model arm, with relative differences in rate ratio interpreted as the percentage of baseline inequity remaining post-intervention, where negative values denote reversal and lower values indicate greater improvement in equity.

**Table 4:** Equity measures for ASMR (per 1000 patients with breast cancer) based on equity axes and model arm

Equity axis	Current care arm		Intervention arm		Differences between arms	
	Rate difference	Rate ratio	Rate difference	Rate ratio	Absolute difference in rate difference	Relative difference in rate ratio
Place of residence	280.38 (259.50; 309.02)	1.39 (1.34; 1.43)	50.72 (30.16; 75.63)	1.08 (1.04; 1.11)	229.65 (198.78; 261.55)	0.20 (0.12; 0.28)
Racial group	381.96 (359.83; 404.00)	1.65 (1.60; 1.69)	271.70 (253.62; 292.72)	1.47 (1.44; 1.52)	110.26 (93.73; 128.13)	0.73 (0.69; 0.77)

ASMR, age-standardised mortality rate.

### Scenario analysis

Scenario analyses showed that the intervention’s cost-effectiveness was influenced by cost structure, mortality assumptions and intervention effectiveness, while equity outcomes were influenced by mortality and effectiveness. Incremental QALYs and LYs declined with increasing discount rates, accompanied by a slight reduction in incremental costs. The intervention was cost-effective at all WTP thresholds at 0% and only at WTP 2 and 3 at 10% (online supplemental additional file 2).

Annuitising costs over 5 years and excluding overdetection costs improved cost-effectiveness, whereas including comprehensive programme costs reduced NMB and eliminated cost-effectiveness at WTP 1.

Using secondary mortality data with higher mortality rates lowered NMB but preserved cost-effectiveness under WHO thresholds while modestly diminishing equity gains. Similarly, assuming persistent year 3 mortality rates reduced health gains yet maintained cost-effectiveness and resulted in relative equity improvements similar to those observed in the base case scenario.

Intervention effect had the strongest influence, with the higher-effectiveness scenario producing the greatest health, economic and equity benefits, including a reversal of rural–urban ASMR inequities, while the lower-effectiveness scenario achieved cost-effectiveness only at the highest threshold, with substantial inequities persisting. Relative differences in rate ratios are interpreted as the percentage of baseline inequity remaining post-intervention, where negative values denote reversal and lower values indicate greater improvement in equity. Tables5  6 provide scenario-specific results for economic and equity outcomes, respectively.

**Table 5:** Comparison of cost-effectiveness model outcomes per person for scenarios explored, presented as mean and 95% CrI

	Baseline case	Lower intervention cost	Higher intervention cost	Secondary mortality data	Alternative mortality extrapolation	Lower intervention effectiveness	Higher intervention effectiveness
Life-years saved	0.356 (0.316;0.391)	0.356 (0.316;0.391)	0.356 (0.316;0.391)	0.153 (0.139;0.215)	0.279 (0.169;0.2	0.114 (0.100;0.129)	0.478 (0.432;0.520)
Incremental QALYs	0.311 (0.277;0.340)	0.311 (0.277;0.340)	0.311 (0.277;0.340)	0.139 (0.123;0.190)	0.243 (0.147; 0.177)	0.099 (0.087;0.112)	0.417 (0.378;0.453)
Incremental Costs (ZAR)	13 513 (13 135;13 861)	2317 (1898;2729)	19 092 (18 711;19 429)	13 795 (13 288;14 398)	13 861 (9112;9665)	12 922 (12 808;13 045)	14 071 (13 559;14 540)
ICER	43 706 (39 340;49 567)	7450 (6236;8817)	61 674 (55 746;69 556)	100 219 (83 341;119 596)	57 222 (53 235;64 200)	131 031 (114 733;149 462)	33 735 (30 393;37 800)
NMB 1 (ZAR)	4487 (2339;6355)	15 584 (14 014;16 803)	−1072 (−3205;774)	−5725 (−6935; −4214)	237 (−1305;1626)	−7172 (−7935; −6342)	10 052 (7573;12 436)
NMB 2 (ZAR)	20 449 (16 570;23 857)	31 435 (28 725;33 938)	14 908 (11 047;18 283)	1432 (−1136;4441)	12 739 (10 103;15 360)	−2073 (−3470; −590)	31 714 (27 024;35 753)
NMB 3 (ZAR)	88 376 (77 126;98 339)	98 888 (91 089;107 115)	82 910 (71 693:92 789)	31 886 (23 560;41 785)	65 941 (58 459;73 803)	19 626 (15 530;23,964)	123 284 (109 850;135 019)

CrI, credible interval; ICER, incremental cost-effectiveness ratio; NMB, net monetary benefit; QALYs, quality-adjusted life-years; ZAR, South African Rand.

**Table 6:** ASMR (per 1000 patients with breast cancer) rate ratios and rate differences for scenario analysis based on equity axis

		Baseline case	Alternative mortality extrapolation	Secondary mortality data	Lower intervention effectiveness	Higher intervention effectiveness
Relative inequality						
Place of residence	Current care arm	1.39 (1.34;1.43)	1.44 (1.37;1.49)	1.34 (1.31;1.38)	1.39 (1.36;1.43)	1.39 (1.36;1.43)
	Intervention arm	1.08 (1.04;1.11)	1.09 (1.07;1.12)	1.21 (1.16;1.24)	1.28 (1.25;1.32)	0.99 (0.96;1.01)
	Relative Difference	0.20 (0.12;0.28)	0.21 (0.17;0.27)	0.61 (0.51;0.70)	0.71 (0.64;0.80)	−0.04 (−0.11;0.03)
Racial group	Current care arm	1.65 (1.60;1.69)	1.62 (1.55;1.69)	1.45 (1.40;1.49)	1.65 (1.60;1.69)	1.65 (1.60;1.69)
	Intervention arm	1.47 (1.44;1.52)	1.48 (1.44;1.52)	1.38 (1.35;1.43)	1.60 (1.56;1.65)	1.42 (1.39;1.46)
	Relative Difference	0.73 (0.69;0.77)	0.78 (0.70;0.89)	0.86 (0.83;0.97)	0.93 (0.89;0.97)	0.64 (0.60;0.68)
Absolute inequality						
Place of residence	Current care arm	280.38 (259.50;309.02)	438.94 (379.49;500.45)	249.26 (230.17;273.52)	280.38 (259.50;309.02)	280.38 (259.50;309.02)
	Intervention arm	50.72 (30.16;75.63)	91.66 (67.38;119.45)	152.18 (122.12;176.32)	199.56 (173.93;228.88)	−10.09 (−30;32;6.96)
	Absolute difference	229.65 (198.78;261.55)	347.28 (290.15;406.36)	97.08 (75.37;120.82)	80.81 (54.49;107.13)	290.47 (256.47;326.87)
Racial group	Current care arm	381.96 (359.83;404.00)	536.39 (501.24;575.04)	285.62 (256.71;311.55)	381.96 (359.83;404.00)	381.96 (359.83;404.00)
	Intervention arm	271.70 (253.62;292.72)	399.50 (378.09;429.76)	244.95 (222.00;268.01)	350.80 (330.53;371.81)	238.31 (221.01;258.77)
	Absolute difference	110.26 (93.73;128.13)	136.89 (89.58;180.75)	40.68 (30.48;50.65)	31.16 (19.74;43.87)	143.65 (126.83;164.67)

ASMR, age-standardised mortality rate.

## Discussion

This analysis demonstrates that the CHW-led educational intervention for breast cancer is likely to be associated with modest gains in LYs and QALYs as well as cost-effective across all three WTP thresholds.

Equity analyses revealed meaningful reductions in inequities by race and place of residence, driven largely by improvements in stage distribution at diagnosis among rural and Black patients. Scenario analyses supported the robustness of these findings, revealing sensitivity of cost-effectiveness results to modelling assumptions and data sources, yet demonstrating consistent robustness in equity impact. The intervention was taken to consistently improve health outcomes and reduce inequity while remaining economically viable under most conditions.

These findings are consistent with the broader literature, which shows that early detection interventions often incur higher upfront costs but generate long-term health gains and cost savings.^35 36^ Although no directly comparable studies of CHW programmes targeting symptomatic presentation were identified, evidence from CHW-led screening and navigation initiatives has demonstrated improvements in early detection, treatment adherence and cost-effectiveness.^37 38^ For example, CHW interventions in the USA improved cancer screening among low-income Latino women, a group disproportionately affected by cancer mortality,^39^ and enhanced screening uptake among Korean American immigrant women, with proven cost-effectiveness.^40^ These studies reinforce the results of the equity analysis from this study, which showed that the intervention could play a meaningful role in reducing inequities linked to race and place of residence in South Africa.

The robustness of this analysis is supported by several methodological strengths. An evidence-based CHW programme was selected as the modelled intervention, building on the success of similar CHW-led initiatives in South Africa for chronic diseases such as diabetes, hypertension and HIV/AIDS, which have been shown to be cost-effective and acceptable in local contexts.^10 11^ Reliability was further enhanced through PSA, comprehensive scenario testing and the application of three alternative WTP thresholds, which is particularly valuable in the absence of an official South African threshold. This approach not only improves the credibility of the findings but also highlights the importance of adopting locally relevant thresholds, as reliance on global benchmarks such as WHO-CHOICE may risk overstating affordability in resource-constrained settings.^41^

Several limitations must also be acknowledged. The capacity of CHWs presents a potential limitation. While breast cancer management accounts for a small share of routine activities, CHWs are frequently working at or beyond capacity, indicating that additional workforce or operational support may be necessary for effective implementation. The intervention effect was derived from a Rwandan study, which may limit generalisability to the South African setting. In addition, the intervention effect was applied uniformly across the model population. It is plausible that the effectiveness of educational interventions may vary across population subgroups, for example according to education level or socioeconomic position. However, insufficient data were available to parameterise subgroup-specific intervention effects. Cost estimates for the intervention were adapted from a breastfeeding programme and may not fully capture the resources required for breast cancer. Mortality estimates required extrapolation beyond the 3-year ABC-DO dataset, and HRQoL values were sourced from China, introducing further cultural and contextual uncertainties. These limitations were addressed by incorporating parameter uncertainty into the model, though future studies using South Africa-specific data would substantially strengthen the evidence base.

Future research should prioritise the generation of high-quality, context-specific data in LMICs, including HRQoL measures, intervention costs and subgroup-level resource use. Research should also explore culturally tailored strategies, such as engaging traditional healers to promote timely referral to improve both intervention acceptability and associated health outcomes.

The demonstrated cost-effectiveness and equity impact of this intervention suggests that it should be considered within national strategies to strengthen early detection and reduce inequities in cancer outcomes. Integration into existing primary healthcare frameworks would enhance accessibility and acceptability, particularly in underserved populations. Its low cost and reliance on existing systems suggest strong potential for implementation. At a policy level, the study underscores the importance of locally appropriate WTP thresholds in economic evaluations given the large variations in NMB values across thresholds, indicating that global benchmarks may not accurately reflect local affordability. More broadly, this work demonstrates how integrating economic evaluation with equity analysis can provide insights into the distributional impacts of targeted interventions. While not intended as a full prioritisation exercise, the findings highlight the potential of such approaches to inform more equitable health policy in South Africa and similar contexts.

## Conclusions

The analyses indicate that the rural CHW educational intervention has the potential to improve health outcomes for South African women with breast cancer. By increasing both LYs and QALYs, the intervention delivers modest health gains, and despite additional costs, it remains cost-effective across all evaluated WTP thresholds, including the base case (~53% of GDP per capita) and the WHO CHOICE thresholds of 1–3 times GDP per capita. Importantly, the intervention also reduces both absolute and relative inequalities in breast cancer outcomes, a benefit sustained across scenario analyses. Taken together, these findings highlight the CHW educational intervention as a highly promising and efficient strategy for improving survival and equity in breast cancer care in rural South Africa.

## Supplementary material

10.1136/bmjopen-2025-114908 online supplemental file 1

10.1136/bmjopen-2025-114908 online supplemental file 2

## Data Availability

Data are available on reasonable request.

## References

[1] South African National Cancer Registry (2019). Cancer in South Africa. South African Natl Cancer Regist.

[2] Bray F, Laversanne M, Sung H (2024). Global cancer statistics 2022: GLOBOCAN estimates of incidence and mortality worldwide for 36 cancers in 185 countries. CA Cancer J Clin.

[3] O’Neil DS, Chen WC, Ayeni O (2019). Breast Cancer Care Quality in South Africa’s Public Health System: An Evaluation Using American Society of Clinical Oncology/National Quality Forum Measures. J Glob Oncol.

[4] Cubasch H, Dickens C, Joffe M (2018). Breast cancer survival in Soweto, Johannesburg, South Africa: A receptor-defined cohort of women diagnosed from 2009 to 11. Cancer Epidemiol.

[5] Joffe M, Ayeni O, Norris SA (2018). Barriers to early presentation of breast cancer among women in Soweto, South Africa. PLoS ONE.

[6] Lambert M, Mendenhall E, Kim AW (2020). Health system experiences of breast cancer survivors in urban South Africa. *Womens Health (Lond Engl*).

[7] Rayne S, Schnippel K, Firnhaber C (2017). Fear of Treatments Surpasses Demographic and Socioeconomic Factors in Affecting Patients With Breast Cancer in Urban South Africa. *JGO*.

[8] Kon ZR, Lackan N (2008). Ethnic disparities in access to care in post-apartheid South Africa. Am J Public Health.

[9] Mapanga W, Norris SA, Craig A (2023). Drivers of disparities in stage at diagnosis among women with breast cancer: South African breast cancers and HIV outcomes cohort. PLoS ONE.

[10] Sahu M, Bayer CJ, Roberts DA (2023). Population health impact, cost-effectiveness, and affordability of community-based HIV treatment and monitoring in South Africa: A health economics modelling study. *PLOS Glob Public Health*.

[11] Gaziano TA, Bertram M, Tollman SM (2014). Hypertension education and adherence in South Africa: a cost-effectiveness analysis of community health workers. BMC Public Health.

[12] Cancer Alliance (2021). Cost of cancer: challenges for the next 10 years. https://canceralliance.org.za/wp-content/uploads/2021/08/Cost-of-Cancer-Advocacy-Report-V1.pdf.

[13] Chanakira EZ, Thomas CV, Balen J (2024). A systematic review of public health interventions to address breast cancer inequalities in low- and middle-income countries. Syst Rev.

[14] Pace LE, Dusengimana JMV, Shulman LN (2019). Cluster Randomized Trial to Facilitate Breast Cancer Early Diagnosis in a Rural District of Rwanda. J Glob Oncol.

[15] National Department of Health (NDoH), Statistics South Africa (Stats SA), South African Medical Research Council (SAMRC) and I (2019). South Africa demographic health survey.

[16] Cancer Research UK (2021). Breast cancer. https://www.cancerresearchuk.org/about-cancer/breast-cancer.

[17] McCormack V, McKenzie F, Foerster M (2020). Breast cancer survival and survival gap apportionment in sub-Saharan Africa (ABC-DO): a prospective cohort study. Lancet Glob Health.

[18] National Cancer Institution (2019). SEER cancer statistics review. 1975-2016.

[19] World Health Organization (2021). Global health observatory data repository. Life tables by country South Africa. https://apps.who.int/gho/data/?theme=main&vid=61540.

[20] International Agency for Research on Cancer (2025). Cancer today. https://gco.iarc.who.int/.

[21] Wilkinson T, Wilkinson M, MacQuilkan K (2021). Health technology assessment methods guide to inform the selection of medicines to the South African national essential medicines list.

[22] Bailey HH, Janssen MF, Varela RO (2021). EQ-5D-5L Population Norms and Health Inequality in Colombia. Value Health Reg Issues.

[23] Wang L, Shi J-F, Zhu J (2018). Health-related quality of life and utility scores of patients with breast neoplasms in China: A multicenter cross-sectional survey. Breast.

[24] Hill H, Kearns B, Duffy S (2022). EE442 The Cost-Effectiveness of Risk Stratified Breast Cancer Screening in the UK. Value Health.

[25] World Bank Group (2025). PPP conversion factor, GDP (LCU per international $) - South Africa.

[26] George G, Mudzingwa T, Horwood C (2020). The cost of the training and supervision of community health workers to improve exclusive breastfeeding amongst mothers in a cluster randomised controlled trial in South Africa. BMC Health Serv Res.

[27] Daviaud E, Subedar H (2012). Staffing norms for primary health care in the context of PHC re-engineering - report to the national department of health. http://www.mrc.ac.za/sites/default/files/files/2016-07-14/StaffingNorms.pdf.

[28] National Department of Health (2017). Breast cancer control policy.

[29] Hassett MJ, Banegas M, Uno H (2019). Spending for Advanced Cancer Diagnoses: Comparing Recurrent Versus De Novo Stage IV Disease. JOP.

[30] The World Bank (2022). GDP per capita (current US$)- South Africa. https://data.worldbank.org/indicator/NY.GDP.PCAP.CD?locations=ZA.

[31] Edoka IP, Stacey NK (2020). Estimating a cost-effectiveness threshold for health care decision-making in South Africa. Health Policy Plan.

[32] Thokala P, Ochalek J, Leech AA (2018). Cost-Effectiveness Thresholds: the Past, the Present and the Future. Pharmacoeconomics.

[33] Public Health and Social Development Sectoral Bargaining Council (2022). Resolution 3 of 2022 - agreement on standardisation of renumeration of community health workers in the department of health.

[34] Joko-Fru WY, Miranda-Filho A, Soerjomataram I (2020). Breast cancer survival in sub-Saharan Africa by age, stage at diagnosis and human development index: A population-based registry study. Int J Cancer.

[35] Hinde S, McKenna C, Whyte S (2015). Modelling the cost-effectiveness of public awareness campaigns for the early detection of non-small-cell lung cancer. Br J Cancer.

[36] Mühlberger N, Sroczynski G, Gogollari A (2021). Cost effectiveness of breast cancer screening and prevention: a systematic review with a focus on risk-adapted strategies. Eur J Health Econ.

[37] Stockdale SE, Keeler E, Duan N (2000). Costs and cost-effectiveness of a church-based intervention to promote mammography screening. Health Serv Res.

[38] Allaire BT, Ekweme D, Hoerger TJ (2019). Cost-effectiveness of patient navigation for breast cancer screening in the National Breast and Cervical Cancer Early Detection Program. Cancer Causes Control.

[39] Mojica CM, Morales-Campos DY, Carmona CM (2016). Breast, Cervical, and Colorectal Cancer Education and Navigation: Results of a Community Health Worker Intervention. Health Promot Pract.

[40] Schuster AL, Frick KD, Huh B-Y (2015). Economic evaluation of a community health worker-led health literacy intervention to promote cancer screening among Korean American women. J Health Care Poor Underserved.

[41] Gloria MAJ, Thavorncharoensap M, Chaikledkaew U (2024). Systematic review of the impact of health care expenditure on health outcome measures: implications for cost-effectiveness thresholds. Expert Rev Pharmacoecon Outcomes Res.

