# Supplementary material for: Equity impact and cost-effectiveness of a community health worker breast cancer educational programme in rural South Africa: a modelling study
Source: BMJ Open. 2026 Apr 21;16(4):e114908. doi: 10.1136/bmjopen-2025-114908 (PMC13110530; doi:10.1136/bmjopen-2025-114908)
Supplement: online supplemental file 1 [file bmjopen-16-4-s001.pdf]

Supplementary Materials

Health Economic Analysis Plan

A formal health economic analysis plan was not developed as a standalone document for this study. However, a conceptual model and a model analysis plan were developed as part of the author's PhD thesis, which guided the structure, assumptions, and analytical approach for the economic evaluation. These materials are fully described and referenced in the thesis(1).

Stakeholder Engagement

Informal stakeholder engagement was conducted, with stakeholders selected to represent a balance of perspectives and insights on breast cancer inequalities and the public health care system in South Africa. The following stakeholder groups were identified: policymakers (1 participant), clinicians (1 participant), advocacy groups (1 participant), and researchers (3 participants).

Two batches of stakeholder engagement sessions were conducted during the project. The first batch aimed to inform the social determinants of breast cancer inequalities, as well as understanding breast management in the South African setting and the associated potential intervention pathways. The second batch aimed to discuss factors affecting model assumptions, as well as validating the resulting conceptual model produced for the project. Engagement took the form of a combination of individual and group sessions, depending on participant availability. The sessions were semi-structured and organised around guiding questions designed to prompt individual reflections as well as encourage discussion among participants. This format balanced consistency across sessions with the flexibility needed to accommodate diverse stakeholder expertise and availability. The primary purpose of these sessions was to inform the model development process by ensuring that key contextual factors were appropriately captured, such as service delivery pathways, current practice, and practical

implementation constraints, so that the model structure, assumptions, and included interventions reflected the realities of the South African health system. These consultations played a critical role in shaping the relevance, feasibility, and credibility of the modelling approach, even though the sessions were not intended to produce generalisable research findings or be formally analysed as qualitative data. Although formal ethics approval was not required for this type of informal, consultative engagement, participants were informed of the purpose of the sessions and their voluntary involvement.

Model Parameters

Mortality Data

All-Cause Mortality

Two appropriate data sources pertaining to breast cancer survival were identified with the first dataset being the ABC-DO dataset containing information on 3-year survival post-diagnosis (2). The second dataset was a population-based registry study that looked at 5-year survival in breast cancer patients in sub-Saharan Africa, with the Eastern Cape registry (South Africa) as one of the registries included in the study (3). The ABC-DO study was selected as the primary dataset, as it provided survival data at substage level using the TNM staging system, as well as information on inequities between the rural and urban population, while the second dataset grouped the cancer stages into early stage and late stage with no information on inequities.

The ABC-DO is a prospective cohort study conducted in five countries across sub-Saharan Africa with South Africa as one of the participating countries (2). The cohort follows 2,156 women with a first time diagnosis of breast cancer and has a 7% loss to follow-up rate over a period of 3 years (2). Baseline crude survival data for the model was obtained from published Kaplan-Meier (KM) curves (Figure 1B) which were available at substage level (i.e. Stages 1,2a,2b,3a,3b,3c,4) (2). Individual patient data was not available from the study and since

information pertaining to the various data coordinates of the KM curves was not available, the published KM curves were reconstructed. A decision was made to digitise all available graphs rather than digitising a single graph and relying solely on the reported hazard ratios, which represent constant relative survival over a three-year period. This approach ensures that time-dependent changes in survival are accurately captured for each subgroup, providing a more detailed and precise analysis. Guyot et al. (2017) proposed an iterative algorithm approach to reconstructing survival curves based on the KM estimation method (iKM) (4). This method was then modified by Liu et al. (2021) resulting in an RShiny interface which was used for reconstruction in this project (5). Digitisation, which was carried out within the app, involved the extraction of raw points pertaining to time and survival probability from the published curves (5). Accuracy was ensured by carefully selecting the maximum number of points and capturing vertical drops at event times. The extracted coordinates were then processed using the modified-iKM algorithm, which reconstructs individual patient data by estimating the number of events and censored cases. The reconstructed data was then validated within the app by comparing it with the original KM curves, assessing summary statistics such as standard error (SE) and conducting survival analyses to ensure reliability (5). Table S1 shows the survival data obtained from digitisation.

Table S1: Survival data obtained through digitisation of KM curves.

	**Time**	**Survival ABC-DO**	**SE**	**95% lower CI**	**95% upper CI**
**Stage 1**	0	1.000			
	1	0.960	0.018	0.927	0.999
	2	0.920	0.027	0.869	0.975
	3	0.850	0.041	0.768	0.930

**Stage 2a**	0	1.000			
	1	0.981	0.008	0.966	0.996
	2	0.895	0.018	0.859	0.931
	3	0.804	0.029	0.750	0.862
**Stage 2b**	0	1.000			
	1	0.907	0.015	0.878	0.937
	2	0.719	0.024	0.674	0.768
	3	0.522	0.030	0.467	0.583
**Stage 3a**	0	1.000			
	1	0.902	0.016	0.871	0.935
	2	0.738	0.025	0.690	0.789
	3	0.530	0.033	0.467	0.596
**Stage 3b**	0	1.000			
	1	0.774	0.020	0.735	0.815
	2	0.546	0.025	0.500	0.596
	3	0.384	0.027	0.334	0.440
**Stage 3c**	0	1.000			
	1	0.674	0.051	0.781	0.326
	2	0.512	0.054	0.629	0.241
	3	0.344	0.053	0.465	0.328
**Stage 4**	0	1.000			
	1	0.500	0.029	0.446	0.561
	2	0.280	0.027	0.232	0.339
	3	0.150	0.025	0.108	0.208

The annual probability of death was calculated using the formula 1 - (S(t) / S(t-1)), as the values obtained from the KM curves were cumulative survival rates. Survival data which was used to

calculate mortality rates was only available for the first 3 years post-diagnosis. Associated 95% confidence intervals were estimated using the calculated probability of death and the numbers at risk provided along with the KM curves in the ABC-DO study (Figure 1B) (2). Uncertainty pertaining to the obtained mortality rates was explored using beta distributions during PSA and was parameterised based on the numbers at risk provided in the ABC-DO study. An assumption was made that mortality rate had a linear decrease from year 4 onwards, until it reached 0 in year 11. Another assumption was made that beyond the 10-year mark, no deaths from breast cancer occurred, with mortality rates returning to the population baseline all-cause mortality rates. These assumptions are supported by clinical evidence and epidemiological data, including long-term survival patterns reported by the Surveillance, Epidemiology, and End Results (SEER) programme in the USA, which demonstrates that breast cancer mortality risk significantly declines several years after diagnosis and approaches that of the general population beyond the 10-year mark (6). The assumption pertaining to mortality extrapolation beyond year 3 was tested during scenario analysis by using a flat mortality rate from year 3 to 10 in the model. Table S2 and Figure S1 illustrate the probabilities of death associated with each stage at diagnosis and time since diagnosis obtained from the digitised curves.

Table S2: Probabilities of all-cause mortality obtained from digitised KM survival curves.

**Time since diagnosis**	**Stage 1 (95% CI)**	**Stage 2a (95% CI)**	**Stage 2b (95% CI)**	**Stage 3a (95% CI)**	**Stage 3b (95% CI)**	**Stage 3c (95% CI)**	**Stage 4 (95% CI)**
**Year 1**	0.040 (0.001, 0.073)	0.019 (0.004, 0.034)	0.093 (0.046, 0.140)	0.098 (0.072, 0.124)	0.224 (0.183, 0.269)	0.326 (0.216, 0.436)	0.500 (0.443, 0.557)
**Year 2**	0.042 (0.005, 0.083)	0.088 (0.056, 0.120)	0.207 (0.141, 0.273)	0.182 (0.148, 0.216)	0.295 (0.249, 0.341)	0.241 (0.141, 0.341)	0.440 (0.383, 0.497)

**Year 3**	0.076 (0.030, 0.134)	0.101 (0.067, 0.135)	0.275 (0.202, 0.348)	0.286 (0.246, 0.326)	0.297 (0.250, 0.344)	0.328 (0.218, 0.438)	0.464 (0.409, 0.523)
**Year 4**	0.072 (0.023, 0.121)	0.089 (0.057, 0.121)	0.240 (0.170, 0.310)	0.250 (0.212, 0.288)	0.260 (0.215, 0.305)	0.287 (0.181, 0.393)	0.408 (0.352, 0.464)
**Year 5**	0.061 (0.016, 0.106)	0.076 (0.046, 0.106)	0.206 (0.140, 0.272)	0.214 (0.178, 0.250)	0.223 (0.181, 0.265)	0.246 (0.145, 0.347)	0.349 (0.295, 0.403)
**Year 6**	0.051 (0.010, 0.092)	0.063 (0.036, 0.090)	0.172 (0.110, 0.234)	0.178 (0.144, 0.212)	0.186 (0.146, 0.226)	0.205 (0.110, 0.300)	0.291 (0.239, 0.343)
**Year 7**	0.041 (0.004, 0.078)	0.051 (0.026, 0.076)	0.137 (0.081, 0.193)	0.143 (0.112, 0.174)	0.149 (0.113, 0.185)	0.164 (0.077, 0.251)	0.233 (0.185, 0.281)
**Year 8**	0.031 (0.000, 0.064)	0.038 (0.016, 0.060)	0.103 (0.053, 0.153)	0.107 (0.080, 0.134)	0.111 (0.079, 0.143)	0.123 (0.046, 0.200)	0.175 (0.132, 0.218)
**Year 9**	0.020 (0.000, 0.046)	0.025 (0.007, 0.043)	0.069 (0.028, 0.110)	0.071 (0.048, 0.094)	0.074 (0.047, 0.101)	0.082 (0.018, 0.146)	0.116 (0.079, 0.153)
**Year 10**	0.010 (0.000, 0.029)	0.013 (0.000, 0.026)	0.034 (0.004, 0.064)	0.036 (0.019, 0.053)	0.037 (0.018, 0.056)	0.041 (0.000, 0.087)	0.058 (0.031, 0.085)

Figure S1: Probability of death based on stage at diagnosis and time since diagnosis.

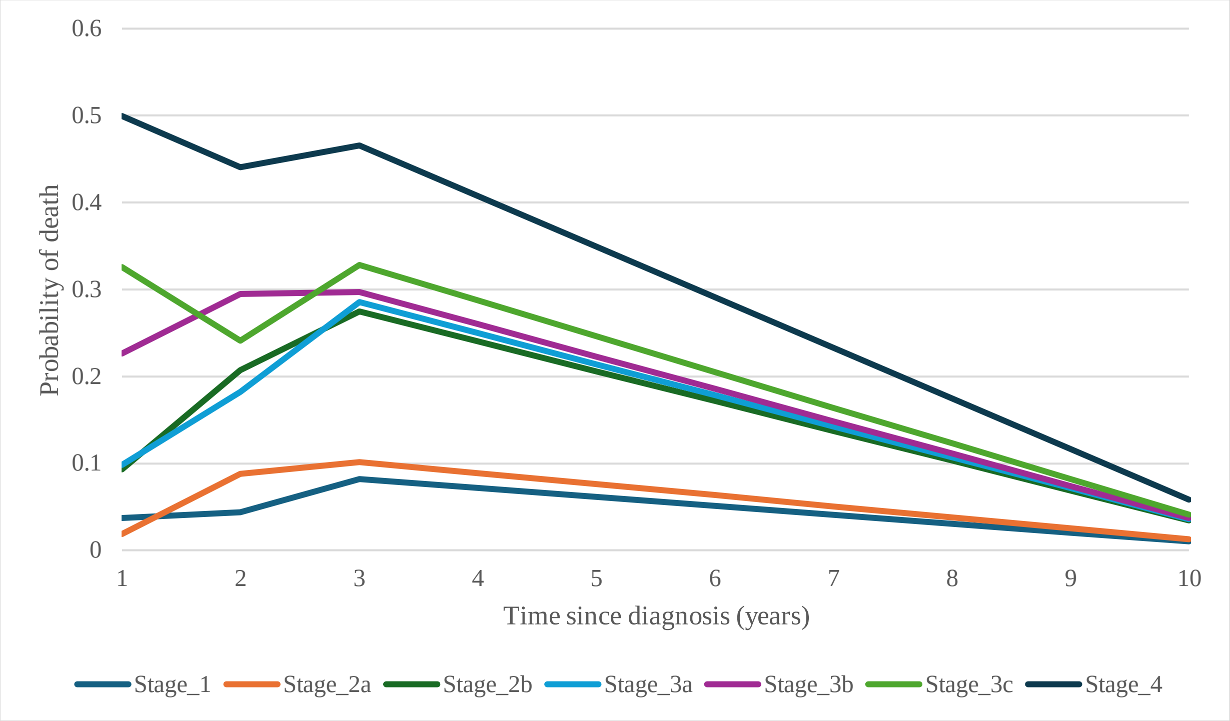

Resulting stage probabilities of death were adjusted to reflect individual risk. The ABC-DO study found that lower survival was associated with rural residence, lower education and socioeconomic status even after adjusting for stage and age at diagnosis (2). Rural residence was associated with a hazard ratio of 1.22 (95% CI: 1.05–1.41) for all-cause mortality, indicating a 22% higher risk of death compared to urban residents. The study also reported hazard ratios by education level, using tertiary education as the reference group: HR = 1.54 (95% CI: 1.22–1.94) for primary education and HR = 1.50 (95% CI: 1.20–1.87) for secondary education (2). Similarly, for socioeconomic position, compared to individuals in the high socioeconomic group, those in the middle group had a HR of 1.19 (95% CI: 0.96–1.47), and those in the low group had a HR of 1.51 (95% CI: 1.20–1.87) (2). The study identified a strong association between lower survival rates and being Black, with Black South African women being 4.25 (95% CI: 1.88-9.65) times as likely to die compared to their White Namibian counterparts (2). Data was not available on survival for White South African women. The

provided survival curves and HRs for race did not account for stage at diagnosis or age (2). A summary of available HRs from the ABC-DO dataset is provided in Table S3.

Table S3: Hazard Ratios for 4-year all-cause mortality from the ABC-DO study (2).

**Variable**	**Category**	**Reference Group**	**Hazard Ratio (HR)**	**95% Confidence Interval (CI)**
**Place of Residence**	Rural	Urban	1.22	1.05 – 1.41
**Education Level**	Primary	Tertiary	1.54	1.22 – 1.94
	Secondary	Tertiary	1.50	1.20 – 1.87
**Socioeconomic Position**	Middle	High	1.19	0.96 – 1.47
	Low	High	1.51	1.20 – 1.87
**Race**	Black South African	White Namibian	4.25	1.88 – 9.65
	Coloured South African	White Namibian	3.02	1.00 – 8.71

Given the relatively short follow-up period of three years, and the use of Cox proportional hazards models in estimating the effects, the reported HRs were considered reasonable approximations of relative risks (7). These will be referred to as such from this point onwards.

No information was available on how SES was defined or calculated in the ABC-DO study, so SES-associated relative risks could not be included in the model. Race-associated relative risks were initially considered but ultimately excluded due to substantial limitations in the available data. The estimates were not adjusted for key confounding factors, including age and stage at diagnosis, which led to implausibly large relative risk values that distorted model outputs. Moreover, the confidence intervals were extremely wide, reflecting high uncertainty in these estimates. The reference category was White Namibian women, with no estimates available for White South African women, further limiting interpretability and applicability. Including these

race-associated relative risks also produced implausible overestimates of survival inequities between rural and urban populations. The exclusion of these risks was therefore based on the combined impact of implausibly large values, high uncertainty, incomplete population coverage, and a high likelihood that the resulting estimates would be inaccurate, rather than simply biased.

This left two equity dimensions for inclusion in the model, which were, place of residence and highest level of education. Input relative risks for both were derived from the study, with the adjusted relative risk associated with rural versus urban residence estimated at 1.22 (95% CI: 1.05–1.41). For education, differences between primary and secondary education were not statistically significant, based on overlapping 95% confidence intervals, so these categories were combined. The input relative risk for the primary/secondary educated group with the tertiary group as reference was estimated to be 1.52 (95% CI: 1.21-1.89). The individual probability of death for each synthetic individual was calculated by multiplying their probability of death by the appropriate relative risks, followed by dividing the resulting value by population-weighted adjustment factors to ensure that overall population mortality remained unchanged after incorporating relative risks. These adjustment factors were calculated as the weighted average of the relative risks associated with each equity axis, ensuring that increased risk in one subgroup (e.g., rural residents) was balanced by lower relative risks in other groups where appropriate (e.g., urban residents). Applying relative risks for both dimensions simultaneously in the model risked over-adjustment and introduced multicollinearity. A calibration exercise was conducted to resolve these issues and to ensure that the model outputs aligned with the expected relative risks.

The calibration process involved applying different relative risk values to adjust the baseline mortality rates, generating corresponding mortality curves. The model-derived output relative

risks were calculated at three years by comparing cumulative mortality rates between groups. Adjustment multipliers were then calculated as the ratio of the expected relative risk to the observed relative risk produced by the model. These multipliers were applied to the initially assumed relative risks, and the process was repeated iteratively until the observed relative risks produced by the model closely matched the expected values. An initial model run using only the relative risk for rural residence (1.22) produced an output relative risk of 1.40, reflecting the additional impact of stage distribution on mortality by place of residence. This value of 1.40 was subsequently used as the target (expected) relative risk for rural residence in the calibration exercise, thereby ensuring that the model appropriately reflected both the assumed risk adjustment and the empirical effects of stage distribution.

Incorporating the relative risk for primary/secondary education required a small adjustment in the input relative risk for the rural population, increasing it slightly to 1.24 (95% CI: 1.07–1.43). This was to maintain the overall output relative risk of 1.40 for the rural population. Adjustment factors were recalculated accordingly to ensure that total survival probabilities across the synthetic population remained consistent with the baseline model using the formulas:

$$adj\_factor_{residence} = \text{proportion urban} + (\text{proportion rural} \times RR_{rural})$$

$$adj\_factor_{education} = \text{proportion tertiary} + (\text{proportion primary/secondary} \times RR_{primary/secondary})$$

Individual probability of death was then calculated based on the appropriate relative risks associated with an individuals' place of residence and highest level of education as below:

$$\frac{Breast\ Cancer\ Mortality_{t,stagex} \times RR_{residence} \times RR_{education}}{adj\_factor_{residence} \times adj\_factor_{education}}$$

By calibrating the model in this way, it was possible to integrate relative risks for place of residence and education while preserving the internal consistency of mortality estimates. The resulting mortality curves provided estimates of survival differences that reflected both the expected equity gradients and the real-world influences of correlated risk factors. The model also produced plausible relative risks for race, with the relative risk for Blacks being 1.47 compared to Whites, and 1.16 for Coloureds compared to Whites. The resulting mortality curves by equity axis are shown in Figures S2, S3, and S4.

Figure S2: All-cause mortality curves by race after applying relative risks.

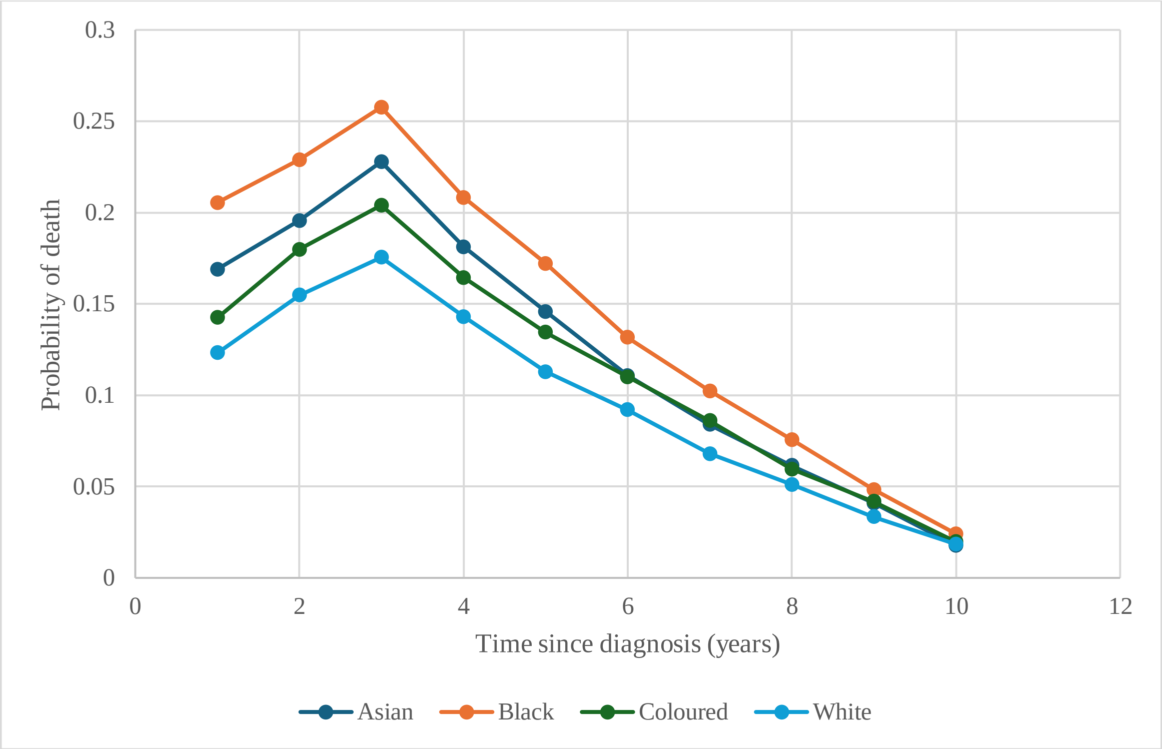

Figure S3: All-cause mortality curves by highest level of education after applying relative risks.

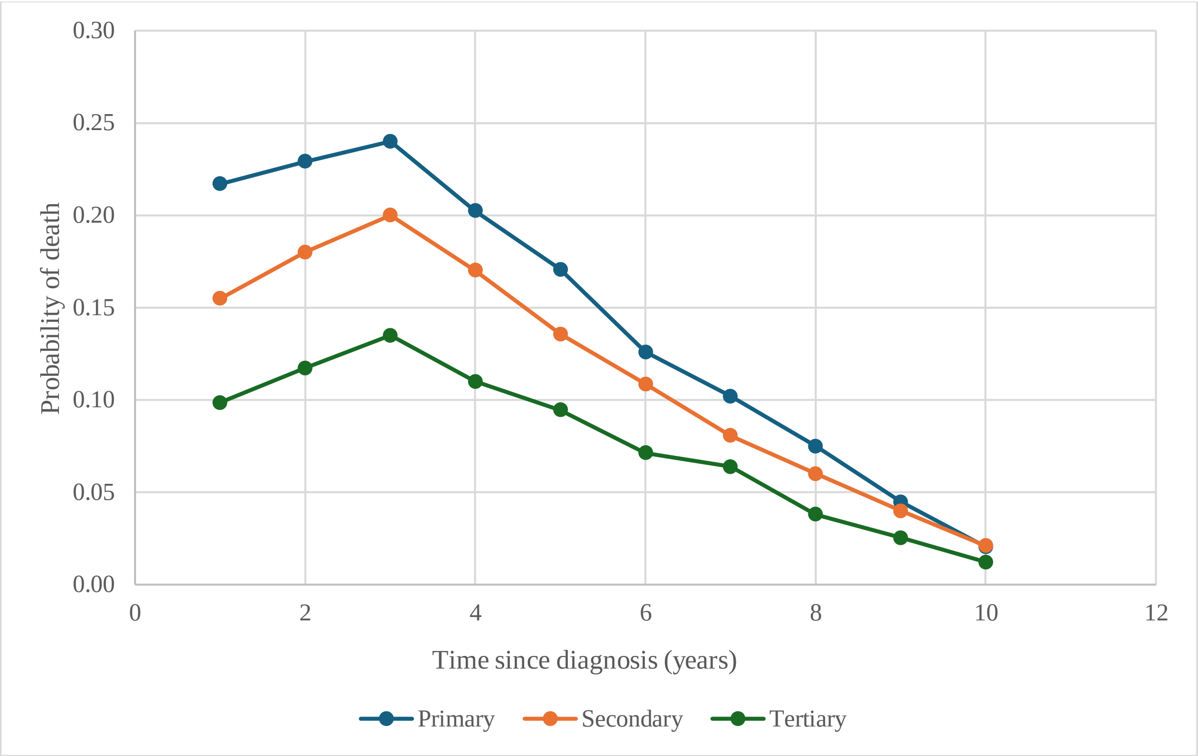

Figure S4: All-cause mortality by place of residence after applying relative risks.

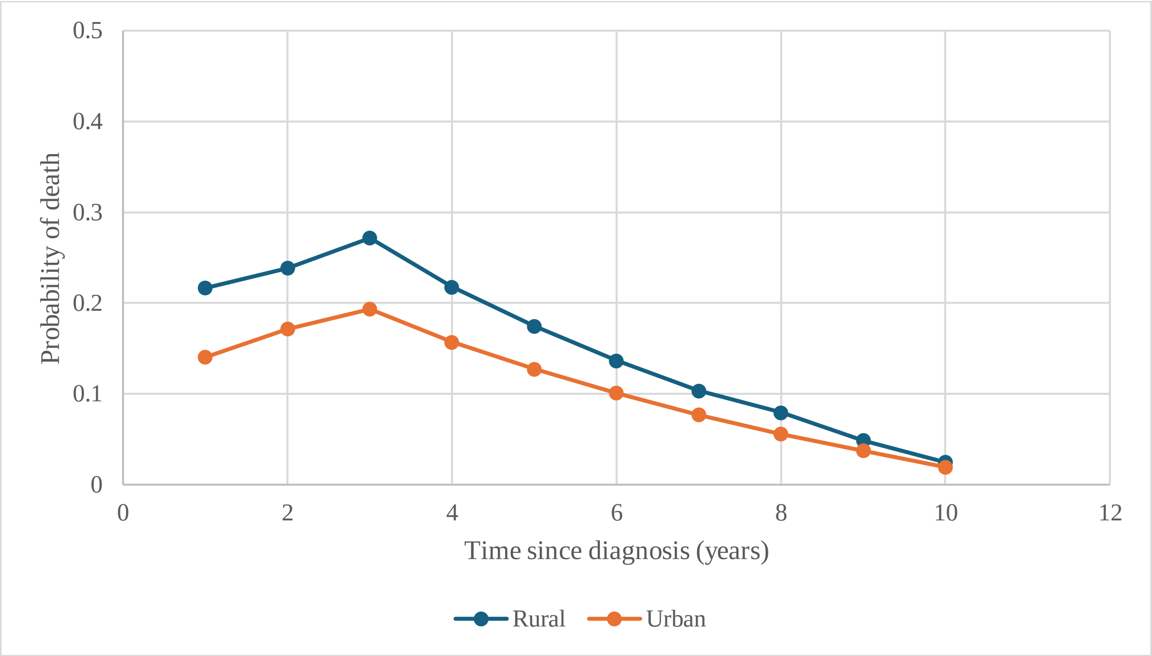

Other-Cause Mortality

Although recent guidelines and comparative studies recommend incorporating general population mortality into survival models, typically through internal additive hazards within the log-likelihood function, this approach was not feasible for this study (8). Implementing such a method requires access to individual-level time-to-event data, detailed cause-specific mortality information, and sufficiently long follow-up to reliably distinguish excess disease-specific mortality from background mortality (8). However, the ABC-DO study provided only crude survival data stratified by TNM substage, without sufficient age-specific detail. This limitation made it difficult to combine the study's all-cause mortality data with external estimates of age-specific mortality from other causes. To address this constraint, a proportion-based competing risks framework was adopted. This approach allowed for the estimation of 10-year probabilities of death from breast cancer versus other causes using available data.

The ABC-DO study provided crude survival rates; therefore, proportions for breast cancer mortality and other cause mortality were calculated for the first 10 years of the model to determine an individual level probability of death from breast cancer versus death from other causes for use in the model.

The probability of dying from other causes by age group was calculated using ASMR which was obtained from the 2019 South African life tables (9), and the 2022 breast cancer ASMR from the Global Cancer Observatory based on national data provided to the WHO (10). The probability of dying from breast cancer in the general female population was subtracted from the probability of dying from all causes to obtain other cause mortality. An assumption was made that other cause mortality is the same in the breast cancer population as in the female population. Individual level random numbers or probabilities were generated between 0 and 1. Individuals with probabilities falling below the threshold for death from other causes were categorised as having died from other causes. Those with probabilities between the threshold

for all-cause mortality and the threshold for death from other causes were assigned to death from breast cancer. A limitation of this approach is that older women in the model exhibit higher other-cause mortality, which is expected, but also lower breast cancer-specific mortality compared to younger women diagnosed at the same stage. This pattern reflects the use of average survival estimates by TNM substage from the ABC-DO study, which do not account for age-specific differences. In the ABC-DO study, age-specific differences in survival, as represented by hazard ratios, generally did not reach statistical significance. The only age group showing a statistically significant difference was participants aged 18–29, with a HR of 1.45 (95% CI: 1.03–2.03). However, as this group represents a small proportion of the overall breast cancer patient population, it is unlikely to materially influence the overall findings. Table S4 shows the values utilised for this calculation, as well as the resulting probabilities. These probabilities were not included in probabilistic sensitivity analysis given that they were obtained from national level data with minimal uncertainty.

Table S4: Estimation of other cause mortality by age in the cohort.

**Age group**	**ASMR general population**	**ASMR for breast cancer in general population**	**Other cause mortality in model**
18-19 years	0.00135	0.00000	0.00135
20-24 years	0.00262	0.00000	0.00262
25-29 years	0.00445	0.00000	0.00445
30-34 years	0.00627	0.00010	0.00617
35-39 years	0.00719	0.00010	0.00709
40-44 years	0.00786	0.00020	0.00766
45-49 years	0.00822	0.00030	0.00792

50-54 years	0.00963	0.00040	0.00923
55-59 years	0.01143	0.00040	0.01103
60-64 years	0.01520	0.00050	0.01470
65-69 years	0.02173	0.00060	0.02113
70-74 years	0.03035	0.00080	0.02955
75-79 years	0.04435	0.00140	0.04295
80-84 years	0.08652	0.00230	0.08422
85+ years	0.18303	0.00350	0.17953

The probability of death from other causes was carried over beyond year 10. Although other cause mortality rates also tend to vary based on socioeconomic status and place of residence, no information was available to allow for the inclusion of these differences within the model beyond year 10, representing a model limitation.

Intervention Effect

To estimate the effect of the intervention on stage at diagnosis, data was utilised from the cluster RCT conducted in Rwanda. The study reported the stage distribution of breast cancer cases in both the intervention and control arms as proportions diagnosed at early stage (stages 1 and 2 combined), stage 3, and stage 4 (shown in Table S5) (11). These proportions were assumed to follow beta distributions within the model to reflect uncertainty around the estimates. Rather than directly applying these proportions to the South African context, a relative impact approach was adopted from a study by Duffy et al. which explored the impact of mammography screening on breast tumour size (12). This decision to apply a relative effect was based on South African Health Technology Assessment guidelines which state that if an intervention is

obtained from a different setting a relative effect measure should be used along with the assumption that relative effects are fully generalisable across jurisdictions (13,14).

An assumption that no one would downstage more than one level was made. Stage distribution in the intervention arm was then compared to that in the control arm to derive the proportion of participants that experienced downstaging. The difference between the proportions for each stage for the intervention and control arm indicated the proportion of individuals who experienced downstaging across stage 3 and 4. This process was conducted within the model to fully capture the uncertainty surrounding intervention effectiveness. Once reallocated, individuals in early stage and stage 3 categories were further subdivided into TNM substages according to the observed baseline distribution among the rural South African breast cancer population as no additional information on post-intervention distribution was available. This allowed the preservation of local substage heterogeneity while incorporating the intervention's estimated effect. Scenario analysis explored an absolute intervention effect, as well as a scenario utilising higher downstaging probabilities for stage 3 and 4. A Dirichlet distribution was used to model uncertainty in the proportions of patients across cancer stages in each arm using the observed counts from the cluster RCT as parameters. The resulting early-stage proportion in the intervention group was used as the ceiling proportion for early-stage cases due to the intervention. Downstaging probabilities were then calculated applying this ceiling effect as well as differences in proportions between the intervention and control groups while ensuring that the resulting probabilities associated with each stage sum to one. Higher effectiveness levels were also explored and were obtained by halving the probability of remaining in the same stage. Figure S5 shows the impact of applying the relative effect downstaging probabilities to the current care stage distribution for the rural population, along with the stage distribution for the urban population in both arms.

Figure S5: Comparison of stage distribution by place of residence before and after the intervention in the modelled population.

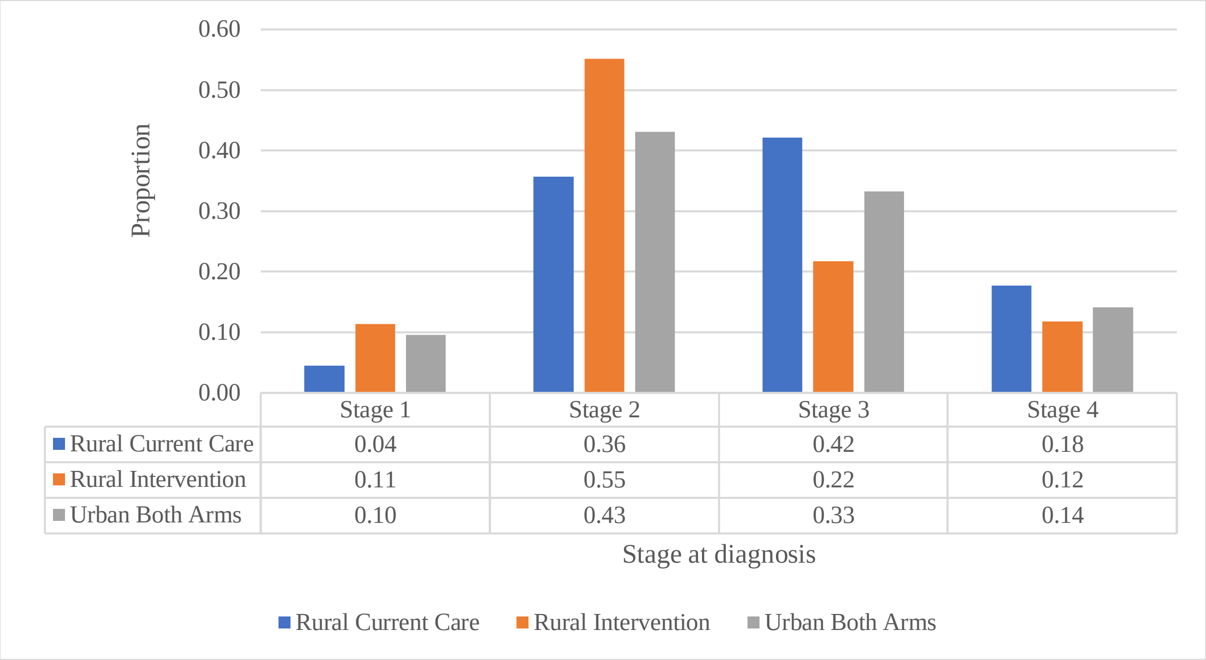

<u>Utility Data</u>

Due to the absence of a South African specific health-related quality of life (HRQoL) dataset, the South African Health Technology Assessment Guidelines allow the use of data from a different country in economic evaluations (14). No datasets depicting population norms were available for sub-Saharan Africa. The HRQoL dataset from Colombia, an upper middle-income country with a high Gini index resembling that of South Africa, was chosen for use in the model as baseline utility values (15). Table S6 shows the estimated baseline utility values applied in the model.

Table S5: Estimated baseline utility values.

Age group	Female Mean index	Female Standard error	Female 95% CI Lower	Female 95% CI Upper
**18-24**	0.973	0.002	0.969	0.977
**25-34**	0.958	0.003	0.952	0.964
**35-44**	0.954	0.004	0.947	0.961
**45-54**	0.933	0.005	0.924	0.942
**55-64**	0.92	0.007	0.906	0.934

Utility multipliers attributed to the reduction in quality of life associated with treatment were calculated using utility data obtained from the most relevant study identified in a systematic review. This study, conducted in China had a larger sample size compared to the other studies identified in the review and presented utility values by disease stage which was essential for incorporation in the model. The study reported an estimated utility of 0.887 (95% CI: 0.875-0.899) in a pre-cancerous population (16), which was used as the baseline utility for calculating multipliers for this study. The utility scores for breast cancer across different stages were reported as follows: 0.789 (95% CI: 0.774-0.805) for stage 1, 0.793 (95% CI 0.783-0.802) for stage 2, 0.774 (95% CI: 0.759-0.788) for stage 3, and 0.686 (95% CI 0.654-0.717) for stage 4. Given the unexpectedly higher utility reported for stage 2 compared to stage 1, a weighted average of the two was used to represent early-stage disease. Utility multipliers for each stage were then calculated by dividing the stage-specific utility score by the estimated utility for the pre-cancer group. The resulting values were applied to the simulated population in the first-year post diagnosis. A post-treatment multiplier calculated from the same study based on a 0.038 increase in utility post-treatment was applied for 4 years post treatment (year 2 to 5). The utility multiplier for stage 4 patients was assumed to remain the same from diagnosis until

death (17). For patients who died from breast cancer, a stage 4 relapse in the final year of life was assumed, therefore the patients were assumed to have stage 4 utility in that final year. After five years, patients were assumed to return to general population baseline utility values, as disutility was considered to be primarily treatment related (17). An exception was made for individuals with stage 4 cancer, for whom a sustained reduction in utility was assumed due to ongoing disease burden. Table S7 shows the resulting utility multipliers. These were modelled using a log-normal distribution within the model due to their strictly positive nature. Proportional changes in utility often result from multiple health impairments or risk factors, which are best represented by multiplicative processes, with the log-normal distribution naturally emerging from such processes (18). Furthermore, since the utility multipliers were derived as ratios, this approach aligns with the well-established behaviour of log-normal distributions in ratio-based variables (18).

Table S6: *Utility adjustment values.*

**Utility adjustment values**	
**Utility multiplier for stage 1 in year 1**	0.8929
**Utility multiplier for stage 2 in year 1**	0.8929
**Utility multiplier for stage 3 in year 1**	0.8726
**Utility multiplier for stage 4 in year 1**	0.7734
**Utility multiplier for stage 1 in year 2 to 5**	0.9357
**Utility multiplier for stage 2 in year 2 to 5**	0.9357
**Utility multiplier for stage 3 in year 2 to 5**	0.9154
**Utility multiplier for stage 4 in year 2 to 5**	0.7734

Cost Data

This section outlines the costs used in the model to evaluate the economic impact of the breast cancer intervention in the South African public health sector. Costs were estimated from the provider perspective and include all relevant healthcare costs associated with diagnosis, staging, treatment, and implementation of the intervention. Costs were stratified by age and cancer stage where applicable. Since diagnosis, staging, and treatment services in the South African public health sector are provided at hospitals, diagnosis at secondary-level facilities and treatment at tertiary-level oncology centres, all located in urban areas while serving both rural and urban populations, the associated costs were assumed to be the same across all population subgroups. While treatment costs could differ due to variations in age distribution, service utilisation, or comorbidity profiles between rural and urban populations as well as Black and non-Black populations, there is no available individual level cost data to accurately quantify these differences. This presents a limitation to the model. Costs associated with over-detection, relapse, and rural-specific intervention components such as CHW training and PHC mentorship were also included.

Breast cancer diagnosis and treatment costs in the South African public health sector were obtained from the HE^2RO arm of the Percept costing model for cancer in South Africa (19,20) The projected rand (ZAR) values for 2023 were used for costs in this model.

Cost of Diagnosis

The average individual cost of diagnosis was presented for individuals below the age of 35 and those 35 and older due to the differences in diagnostic procedure for these groups in the National Breast Cancer Policy (21), with women aged 35 and over receiving a mammogram in addition to CBE, ultrasound and core biopsy (19,20). An obligatory follow up/results visit was also included in the cost of diagnosis (19,20). The aggregate cost of diagnosis for individuals

under the age of 35 was reported in the Percept costing model to be ZAR 6,111 (I$836) while that for individuals 35 and over was reported to be ZAR 7,353 (I$1,006).

Cost of over-detection

The cost associated with over-detection which is bound to occur due to increased awareness in the population was also included for the rural population in the intervention arm. In the cluster RCT intervention study, for every breast cancer diagnosis in the intervention arm, 2.82 (95% CI: 2.71-2.93) additional women underwent the diagnostic process without breast cancer, in comparison to the control arm (11). For the model, the cost of diagnosis for each rural woman in the intervention arm was multiplied by 3.82 (95% CI: 3.71-3.93) to account for the costs associated with over-detection. This value was assumed to follow a gamma distribution within the model. Model sensitivity to this cost was tested by excluding it from intervention costs during scenario analysis as part of a scenario that explored a lower intervention cost.

Cost of staging

The cost of staging included tests such as chest x-ray, liver ultrasonography, full blood count, urea and electrolyte test, enzyme tests as well as a full metabolic profile with bone scans administered when deemed necessary (in approximately 7.5% of the patient population) (19,20). The cost of staging was ZAR 4,908 (I$671). This cost was only applied to confirmed cancer cases.

Cost of treatment

An assumption that the cost of treatment was incurred in the first-year post diagnosis was made in the model, based on guidelines from the National Breast Cancer Control Policy (2017) (21). An average individual cost of treatment based on the stage at diagnosis was calculated in the HE^2RO costing model. The HE^2RO costing model assumed that 50% of stage 1 cases received

a lumpectomy while the rest received a mastectomy (19,20). The proportion of all cancer patients receiving endocrine therapy was assumed to be 81% (19). All stage 2, 3, and 4 patients in the HE^{2}RO costing model received radiotherapy along with chemotherapy (19,20). All stage 2 and 3 patients were assumed to receive a mastectomy and 50% of stage 4 patients were assumed to receive palliation (19,20). These above assumptions are based on the HE^{2}RO costing model and do not reflect any new assumptions introduced in this project.

For this project, an assumption was made that if a patient dies from cancer, they experience a stage 4 relapse in the year preceding their death. This relapse is assumed to incur a diagnostic cost and a proportion (0.5781) of the stage 4 treatment cost, based on findings from a study that looked at the difference in treatment costs for a first-time diagnosis and a recurrent diagnosis (22). Patients who die in the first year of the model did not receive any relapse-related costs based on an assumption that death would have occurred during the initial treatment period.

Table S7: Treatment costs.

**Total costs of treatment per patient**	
**Stage 1**	ZAR 94,115 (I$12,875)
**Stage 2-3**	ZAR 105,160 (I$14,386)
**Stage 4**	ZAR 110,689 (I$15,142)
**Relapse treatment**	ZAR 63,989 (I$8,754)

Cost of intervention

Components of the intervention that are currently not available in the South African public health sector as current care were considered for inclusion in determining the intervention cost for the project. These were costs associated with the training of CHWs and mentorship at PHC

level by trained midwives (11). Intervention costs were separated into cost of initial training/ diagnosed rural cancer case and cost of human resources/ diagnosed rural cancer.

CHW related costs

In the RCT study conducted in Rwanda over a 2 year period, a ratio of approximately 53 trained CHWs per diagnosed breast cancer case was observed (11,23). This ratio was used to estimate the CHW costs for the intervention. The cost of providing initial training for CHWs was estimated based on a costing study that looked at the impact of providing training to CHWs in rural South Africa for the management of breastfeeding women (24). This was due to the absence of context specific studies that explored breast cancer awareness interventions. The cost of training for the breast feeding study, included the cost of transportation, training venue, accommodation, and catering costs (24). A daily cost of training for the 53 CHWs required for a breast cancer diagnosis was estimated to be ZAR 1,720 (I$325) in South African Rands 2013 value. The cost was then converted to its 2023 value using a consumer price index (CPI) of 90.132 for 2013 and 150.647 for 2023 to obtain the cost of training for the intervention, resulting in a cost of ZAR 2,875 (I$393)(25).

Based on standard practice and prescribed duties of CHWs in South Africa, the time they are likely to spend dealing with breast cancer related issues within the community is limited. A significant portion of their catchment population includes children and men, who are at minimal risk for breast cancer. As a result, CHWs are expected to spend only a small fraction of their time on breast cancer outreach, therefore an assumption was made that the associated costs are negligible. This assumption was tested during scenario analyses, with a higher cost scenario that incorporated a dedicated 1% cost of CHW time to the intervention being explored.

Midwife Mentorship Costs

The number of PHCs in rural South Africa was determined to be 1,696 through stakeholder engagement. The PHC cost per rural diagnosed case receiving the intervention was determined by first estimating the number of diagnosed cases in the population. This was done using an estimate of the 2023 breast cancer incidence (17,461) as determined by the HE^{2}RO incidence and costing report, together with an estimated proportion of those patients expected to be from rural areas (0.356) obtained from the DHS survey (2016) (26). This resulted in 5,336 breast cancer cases.

Based on the staffing norms in South Africa, whereby each district is expected to have 1 advanced midwife, an assumption was made that a single full-time midwife can be employed per district to provide supervision, resulting in 52 full-time midwife positions (27). An average cost to employer of ZAR 491,487 (I$67,235) per midwife per annum was obtained from the South African government annual pay schedule for 2023. The overall amount for the 52 midwives was ZAR 25,557,246 (I$3,496,203). This was then divided by the estimated 2023 rural breast cancer incidence to get a cost per rural diagnosed case of ZAR 4,790 (I$655).

The overall mean cost/rural diagnosed case was then calculated as the sum of CHW related costs and midwife mentorship costs. This resulted in a cost of ZAR 7,665 (I$1,049) per rural diagnosed case. In the base case scenario, annuitisation was not carried out as an assumption of a single year intervention was made. Table S9 shows a summary of costs associated with the intervention.

Table S8: Summary of intervention costs.

**Intervention Component**	**Cost per rural diagnosed case**
Community Healthcare Worker training	ZAR 2,875 (I$393)

Cost of mid-wife mentorship for PHC nurses	ZAR 4,790 (I$655)
**Total Cost per rural diagnosed case**	**ZAR 7,665 (I$1,049)**

Scenario Analyses

Scenario analyses were conducted to examine structural and methodological uncertainties beyond the parameter uncertainty addressed in the base case. Scenario selection was guided by a structured consideration of key factors deemed to significantly influence the outcomes of interest. Scenarios pertaining to variables that were considered to have high levels of uncertainty and impact on model outcomes were considered. The explored scenarios were as follows:

- A lower intervention cost scenario was explored. It looked at the impact of a 5% annuitisation rate on the costs associated with intervention training, and the exclusion of diagnostic costs associated with individuals who presented for assessment but did not have breast cancer. In this scenario, training costs were spread over a 5-year period, based on the assumption that the knowledge and skills gained by CHWs would remain effective for at least five years, with retraining required thereafter. In contrast, the base case did not apply annuitisation to training costs. This was due to the inherent uncertainty around the duration of effectiveness of educational interventions, which are not physical assets and therefore do not have clearly defined lifespans. The longevity of training benefits may vary depending on factors such as staff turnover, changes in community health priorities, and the availability of ongoing support or supervision. As a result, it is difficult to justify a specific annuitisation period in the base case without strong empirical evidence. However, because the treatment of training costs can materially influence the cost-effectiveness of the intervention, this represents a key

structural uncertainty. This scenario also excluded the diagnostic costs associated with individuals who present for assessment but do not have breast cancer. This cost was included in the base case to fully reflect the health system impact of increased community awareness and presentation rates. Its exclusion in this scenario reflects an alternative assumption where only costs directly tied to breast cancer diagnosis and treatment are considered. This variation tests the sensitivity of the model to potential overestimation of diagnostic resource use, particularly in situations where non-cancer referrals might be triaged earlier or managed at lower cost.

- A higher cost scenario was explored to capture additional resource implications that were not included in the base case intervention cost. This scenario incorporated three key additional cost components: firstly, allocating 1% of CHW time specifically to the dissemination of breast cancer knowledge, resulting in an additional cost of ZAR 536 (I$73) per rural diagnosed case (28). While this represents a small proportion of CHW time, it acknowledges the opportunity cost associated with diverting CHWs from other duties, such as routine health promotion or support for chronic disease management. Secondly, the costs associated with providing a weekly half-day breast clinic at PHC facilities were also included. While this component was excluded from the base case due to the relatively low incidence of breast cancer in rural areas compared to the number of PHCs, making routine breast clinics at each facility potentially inefficient, it was included in this scenario to reflect a more comprehensive and resource-intensive delivery model. The rationale for this inclusion was to test the sensitivity of cost-effectiveness outcomes to the potential scaling-up of dedicated services should there be need. It was assumed that one PHC nurse would staff each breast clinic due to the relatively low incidence of breast cancer in rural areas compared to the total number of PHCs (1,696). Using the 2023 South African government pay schedule, the annual

salary cost per nurse was estimated at ZAR 55,110 (I$7,539), translating to a total annual cost of ZAR 93,467,320 (I$12,786,227) for all PHCs. Based on the estimated 2023 rural breast cancer incidence of 5,336 cases, this amounted to an additional cost per diagnosed rural case of ZAR 17,516 (I$2,396). Thirdly, additional follow-up costs for patients diagnosed as not having breast cancer were included, assuming each such patient would require one follow-up visit costing ZAR 162 per visit (29). This resulted in an additional cost of ZAR 457(I$63) per rural diagnosed case considering that an additional 2.82 patients were observed to present with breast symptoms but no cancer in the intervention study. Table S9 provides a breakdown of the additional costs and the resulting overall cost for scenario analysis.

Table S9: Costs associated with the higher intervention cost scenario.

**Intervention Component**	**Cost per rural diagnosed case**
Base case intervention cost per rural diagnosed case	ZAR7,665(I$1,049)
1% CHW dissemination time	ZAR536 (I$73)
Weekly breast clinic at PHC level	ZAR17,516 (I$2,396)
Follow up cost for those with breast symptoms without breast cancer	ZAR457 (I$63)
**Overall cost per diagnosed case for scenario analysis**	**ZAR26,174(I$3,581)**

- A scenario was explored that used secondary mortality data obtained from a population-based registry study examining 5-year breast cancer survival across sub-Saharan Africa, which included the Eastern Cape registry in South Africa (3). This scenario modelled

higher mortality rates by stratifying outcomes according to early- and late-stage disease at diagnosis. The rationale for incorporating this alternative mortality source was to test sensitivity of the model to different survival assumptions, particularly in the context of limited, high-quality, stage-specific mortality data. For this scenario, a calibration exercise was carried out to ensure that the relative risks used to adjust all-cause mortality resulted in the expected levels of inequality by place of residence and highest level of education. The resulting input relative risks were 1.33 (95% CI:1.14-1.54) for rural place of residence and 1.63 (95% CI:1.30-2.04) for primary/secondary highest level of education. Table S10 shows all-cause mortality associated with this scenario.

Table S10: All-cause mortality in breast cancer population obtained from a secondary source for scenario analysis (3).

	**Early Stage (Stage 1 and 2) (95% CI)**	**Late Stage (Stage 3 and 4) (95% CI)**
**Year 1**	0.076 (0.051; 0.101)	0.178 (0.156; 0.200)
**Year 2**	0.100 (0.072; 0.128)	0.251 (0.225; 0.277)
**Year 3**	0.110 (0.081; 0.139)	0.220 (0.195; 0.245)
**Year 4**	0.098 (0.071; 0.125)	0.182 (0.158; 0.206)
**Year 5**	0.068 (0.045; 0.091)	0.068 (0.052; 0.084)
**Year 6**	0.057 (0.036; 0.078)	0.057 (0.043; 0.071)
**Year 7**	0.045 (0.027; 0.063)	0.045 (0.033; 0.057)
**Year 8**	0.034 (0.019; 0.049)	0.034 (0.023; 0.045)
**Year 9**	0.023 (0.011; 0.035)	0.023 (0.014; 0.032)
**Year 10**	0.011 (0.003; 0.019)	0.011 (0.005; 0.017)

- An alternative scenario was explored in which the breast cancer mortality rate remained constant from year 3 to year 10, rather than continuing to decline linearly as assumed in the base case analysis. This scenario was included to test sensitivity of the model to the long-term mortality trend assumption. The constant mortality scenario represents a more conservative assumption, acknowledging the possibility that early gains in mortality reduction may plateau after the initial years following diagnosis and treatment.

Table S11 : Alternative extrapolation of all-cause mortality rates in breast cancer population.

	**Stage 1**	**Stage 2a**	**Stage 2b**	**Stage 3a**	**Stage 3b**	**Stage 3c**	**Stage 4**
**Year 1**	0.040<br>(0.001, 0.073)	0.019<br>(0.004, 0.034)	0.093<br>(0.046, 0.140)	0.098<br>(0.072, 0.124)	0.224<br>(0.183, 0.269)	0.326<br>(0.216, 0.436)	0.500<br>(0.443, 0.557)
**Year 2**	0.042<br>(0.005, 0.083)	0.088<br>(0.056, 0.120)	0.207<br>(0.141, 0.273)	0.182<br>(0.148, 0.216)	0.295<br>(0.249, 0.341)	0.241<br>(0.141, 0.341)	0.440<br>(0.383, 0.497)
**Year 3 -10**	0.076<br>(0.030, 0.134)	0.101<br>(0.067, 0.135)	0.275<br>(0.202, 0.348)	0.286<br>(0.246, 0.326)	0.297<br>(0.250, 0.344)	0.328<br>(0.218, 0.438)	0.464<br>(0.409, 0.523)

- Alternative levels of intervention effectiveness beyond PSA, due to the high levels of uncertainty associated with the intervention, as the intervention study was conducted in a different setting. A more conservative estimate of the intervention effect was explored by applying an absolute effect approach. Post-intervention stage distributions and their associated uncertainty were derived using a Dirichlet distribution, based on the assumption that the observed distribution in the intervention arm represented the maximum (ceiling) achievable intervention effect. The resulting stage distributions were then used in the PSA. Higher effectiveness levels were also explored and were obtained by halving the probability of remaining in the same stage.

Table S12: Alternative effectiveness levels for the intervention (11).

Downstaging probabilities			
	**Early Stage**	**Stage 3**	**Stage 4**
**Probability of staying in same stage – lower effectiveness**	1.000	0.823	0.896
**Probability of downstaging – lower effectiveness**	0.000	0.177	0.104
**Probability of staying in same stage – baseline analysis**	1.000	0.375	0.667
**Probability of downstaging – baseline analysis**	0.000	0.625	0.333
**Probability of staying in same stage – higher effectiveness**	1.000	0.188	0.333
**Probability of downstaging – higher effectiveness**	0.000	0.812	0.667

Table S13: Parameter Distributions and Sources for PSA.

**Parameter group**	**Parameter Description**	**Mean**	**95% CI**	**Distribution type**	**Distribution parameters**		**Source**
Proportion with cancer stage	Proportion with stage 1	0.057	(0.047; 0.062)	Beta	200	3283	SABC Dataset (26)
Proportion with cancer stage	Proportion with stage 2	0.280	(0.266; 0.295)	Beta	1032	2651	SABC Dataset (26)
Proportion with cancer stage	Proportion with early stage	0.338	(0.319; 0.3450)	Beta	1232	2451	SABC Dataset(26)
Proportion with cancer stage	Proportion of early-stage cancers that are stage 1	0.170	(0.149; 0.191)	Beta	209	1023	SABC Dataset (26)
Proportion with cancer stage	Proportion of early-stage cancers that are stage 2a	0.423	(0.395; 0.451)	Beta	521	711	SABC Dataset (26)
Proportion with cancer stage	Proportion of early-stage cancers that are stage 2b	0.407	(0.379; 0.434)	Beta	501	731	SABC Dataset(26)

Proportion with cancer stage	Proportion with stage 3	0.460	(0.444; 0.477)	Beta	1696	1987	SABC Dataset (26)
Proportion with cancer stage	Proportion with stage 4	0.202	(0.189; 0.215)	Beta	744	2939	SABC Dataset (26)
Proportion with cancer stage	Proportion of stage 2 cancers that are 2a	0.510	(0.479; 0.540)	Beta	526	506	SABC Dataset (26)
Proportion with cancer stage	Proportion of stage 2 cancers that are 2b	0.490	(0.460; 0.521)	Beta	506	526	SABC Dataset (26)
Proportion with cancer stage	Proportion of stage 3 cancers that are 3a	0.375	(0.351; 0.397)	Beta	635	1061	SABC Dataset (26)
Proportion with cancer stage	Proportion of stage 3 cancers that are 3b	0.530	(0.506; 0.553)	Beta	898	798	SABC Dataset (26)
Proportion with cancer stage	Proportion of stage 3 cancers that are 3c	0.096	(0.082; 0.110)	Beta	162	1534	SABC Dataset (26)
Utility	Utility multiplier for stage 1 patients in year 1	0.8929	(0.8869; 0.8988)	Lognormal	-0.11328	0.01	HRQoL in Chinese population (16)

Utility	Utility multiplier for stage 2 patients in year 1	0.8929	(0.8897; 0.8988)	Lognormal	-0.11328	0.01	HRQoL in Chinese population (16)
Utility	Utility multiplier for stage 3 patients in year 1	0.8726	(0.8674; 0.8765)	Lognormal	-0.13628	0.01	HRQoL in Chinese population (16)
Utility	Utility multiplier for stage 4 patients in year 1	0.7734	(0.7474; 0.7976)	Lognormal	-0.25696	0.08	HRQoL in Chinese population (16)
Utility	Utility multiplier post treatment for stage 1	0.9357	(0.9304; 0.9822)	Lognormal	-0.06646	0.01	HRQoL in Chinese population (16)
Utility	Utility multiplier post treatment for stage 2	0.9357	(0.9304; 0.9822)	Lognormal	-0.06646	0.01	HRQoL in Chinese population (16)
Utility	Utility multiplier post treatment for stage 3	0.9154	(0.8994; 0.9599)	Lognormal	-0.08839	0.01	HRQoL in Chinese population (16)
Utility	Utility multiplier post treatment for stage 4	0.8162	(0.7794; 0.881)	Lognormal	-0.2031	0.08	HRQoL in Chinese population (16)
Cost	Cost of diagnosis for women below the age of 35	6110.501	-	Gamma	100	122.21	Percept model (19)

Cost	Cost of diagnosis for women above the age of 35	7352.947	-	Gamma	100	147.06	Percept model (19)
Cost	Cost of staging	4907.29	-	Gamma	100	98.15	Percept model (19)
Cost	Cost of treating stage 1 cancer	118818	-	Gamma	100	2376.36	Percept model (19)
Cost	Cost of treating stage 2 cancer	132761	-	Gamma	100	2655.22	Percept model (19)
Cost	Cost of treating stage 3 cancer	132761	-	Gamma	100	2655.22	Percept model (19)
Cost	Cost of treating stage 4 cancer	139742	-	Gamma	100	2794.84	Percept model (19)
Cost	Cost of palliation	63989	-	Gamma	100	1279.78	Percept model (19)
Cost	Human resources costs at PHC level	4789	-	Gamma	100	95.78	Salary Schedules South Africa
Cost	Cost of CHW training	2875.39	-	Gamma	100	57.51	Costing of breastfeeding intervention (24)
Cost	Cost multiplier for over-detection	3.82	(3.71; 3.93)	Gamma	100	0.0382	Intervention study, Rwanda RCT (11)

Intervention effect (relative)	Proportion of early-stage cases in control group	0.22	-	Beta	2	7	Intervention study, Rwanda RCT (11)
Intervention effect (relative)	Proportion of early-stage cases in intervention group	0.5	-	Beta	9	9	Intervention study, Rwanda RCT (11)
Intervention effect (relative)	Proportion of stage 3 cases in control group	0.44	-	Beta	4	5	Intervention study, Rwanda RCT (11)
Intervention effect (relative)	Proportion of stage 3 cases in intervention group	0.28	-	Beta	5	13	Intervention study, Rwanda RCT (11)
Intervention effect (relative)	Proportion of stage 4 cases in control group	0.33	-	Beta	3	6	Intervention study, Rwanda RCT (11)
Intervention effect (relative)	Proportion of stage 4 cases in intervention group	0.22	-	Beta	4	14	Intervention study, Rwanda RCT (11)
Intervention effect (absolute effect)	Proportion of early-stage cases in intervention group	0.475	(0.281; 0.686)	Beta	10.51	11.64	Intervention study, Rwanda RCT (11)
Intervention effect (absolute effect)	Proportion of early-stage cases in control group	0.250	(0.056;0.499)	Beta	3.59	10.72	Intervention study, Rwanda RCT (11)

Intervention effect (absolute effect)	Proportion of stage 3 cases in intervention group	0.287	(0.114; 0.502)	Beta	5.42	13.52	Intervention study, Rwanda RCT (11)
Intervention effect (absolute effect)	Proportion of stage 3 cases in control group	0.415	(0.177;0.705)	Beta	6.40	9.02	Intervention study, Rwanda RCT (11)
Intervention effect (absolute effect)	Proportion of stage 4 cases in intervention group	0.238	(0.083; 0.432)	Beta	4.31	13.81	Intervention study, Rwanda RCT (11)
Intervention effect (absolute effect)	Proportion of stage 4 cases in control group	0.334	(0.119;0.601)	Beta	5.00	9.97	Intervention study, Rwanda RCT (11)
Inequities in mortality	Relative risk of death in rural population with urban reference	1.24	(1.07; 1.43)	Lognormal	0.21	0.07	ABC-DO survival study (2)
Inequities in mortality	Adjusted relative risk of death in population with primary or secondary education with tertiary education as reference	1.52	(1.21-1.89)	Lognormal	-0.42	0.11	ABC-DO survival study (2)

References

1. Chanakira EZ. Addressing breast cancer inequalities in the South African public health sector: a modelling approach in a LMIC setting [Internet]. University of Sheffield; 2024. Available from: https://etheses.whiterose.ac.uk/id/eprint/37568/

2. McCormack V, McKenzie F, Foerster M, Zietsman A, Galukande M, Adisa C, et al. Breast cancer survival and survival gap apportionment in sub-Saharan Africa (ABC-DO): a prospective cohort study. Lancet Glob Heal. 2020;8(9):e1203–12.

3. Joko-Fru WY, Miranda-Filho A, Soerjomataram I, Egue M, Akele-Akpo MT, N'da G, et al. Breast cancer survival in sub-Saharan Africa by age, stage at diagnosis and human development index: A population-based registry study. Int J Cancer. 2020;146(5):1208–18.

4. Guyot P, Ades AE, Beasley M, Lueza B, Pignon JP, Welton NJ. Extrapolation of Survival Curves from Cancer Trials Using External Information. Med Decis Mak. 2017;37(4):353–66.

5. Liu N, Zhou Y, Lee JJ. IPDfromKM: reconstruct individual patient data from published Kaplan-Meier survival curves. BMC Med Res Methodol. 2021;21(1):1–22.

6. National Cancer Institution. SEER Cancer Statistics Review. 1975-2016. SEER website. 2019.

7. Austin PC. Absolute risk reductions and numbers needed to treat can be obtained from adjusted survival models for time-to-event outcomes. J Clin Epidemiol [Internet]. 2010;63(1):46–55. Available from: https://www.sciencedirect.com/science/article/pii/S089543560900095X

8. van Oostrum I, Ouwens M, Remiro-Azócar A, Baio G, Postma MJ, Buskens E, et al. Comparison of Parametric Survival Extrapolation Approaches Incorporating General Population Mortality for Adequate Health Technology Assessment of New Oncology Drugs. Value Heal [Internet]. 2021;24(9):1294–301. Available from: https://doi.org/10.1016/j.jval.2021.03.008

9. World Health Organization. Life tables by country South Africa [Internet]. Global Health Observatory data repository. 2021 [cited 2023 Aug 21]. Available from: https://apps.who.int/gho/data/?theme=main&vid=61540

10. International Agency for Research on Cancer. Cancer Today [Internet]. 2025 [cited 2025 Feb 13]. Available from: https://gco.iarc.who.int/

11. Pace LE, Dusengimana JMV, Shulman LN, Schleimer LE, Shyirambere C, Rusangwa C, et al. Cluster randomized trial to facilitate breast cancer early diagnosis in a rural district of Rwanda. J Glob Oncol. 2019;2019(5):1–13.

12. Duffy SW, Tabar L, Vitak B, Warwick J. Tumor size and breast cancer detection: What might be the effect of a less sensitive screening tool than mammography? Breast J. 2006 Jan;12(SUPPL. 1).

13. Drummond M, Barbieri M, Cook J, Glick HA, Lis J, Malik F, et al. Transferability of economic evaluations across jurisdictions: ISPOR good research practices task force report. Value Heal [Internet]. 2009;12(4):409–18. Available from: http://dx.doi.org/10.1111/j.1524-4733.2008.00489.x

14. Wilkinson T, Wilkinson M, MacQuilkan K. Health Technology Assessment Methods Guide To Inform the Selection of Medicines To the South African National Essential Medicines List. 2021;(June).

15. Bailey HH, Janssen MF, Varela RO, Moreno JA. EQ-5D-5L Population Norms and Health Inequality in Colombia. Value Heal Reg Issues [Internet]. 2021;26:24–32. Available from: https://doi.org/10.1016/j.vhri.2020.12.002

16. Wang L, Shi JF, Zhu J, Huang HY, Bai YN, Liu GX, et al. Health-related quality of life and utility scores of patients with breast neoplasms in China: A multicenter cross-sectional survey. Breast. 2018;39:53–62.

17. Hill H, Kearns B, Duffy S. The Cost-Effectiveness of Risk Stratified Breast Cancer Screening in the UK. 2022.

18. Limpert E, Stahel WA, Abbt M. Log-normal distributions across the sciences: Keys and clues. Bioscience. 2001;51(5):341–52.

19. Cancer Alliance. Estimating and projecting the burden of cancer in South Africa [Internet]. 2021. Available from: https://canceralliance.org.za/wp-content/uploads/2021/08/Percept-report-on-the-Cost-of-Cancer-in-South-Africa-v1.pdf

20. Percept Actuaries and Consultants. Cost of Cancer Model. 2020.

21. National Department of Health. Breast cancer control policy. Natl Dep Heal. 2017;(June):1–68.

22. Hassett MJ, Banegas M, Uno H, Weng S, Cronin AM, O'Keeffe Rosetti M, et al. Spending for Advanced Cancer Diagnoses: Comparing Recurrent Versus De Novo Stage IV Disease. J Oncol Pract. 2019;15(7):e616–27.

23. Dusengimana JMV, Keating NL, Hategekimana V, Rugema V, Bigirimana JB, Costas-Chavarri A, et al. Impact of breast cancer early detection training on Rwandan health workers' knowledge and skills. J Glob Oncol. 2018;2018(4):1–10.

24. George G, Mudzingwa T, Horwood C. The cost of the training and supervision of

community health workers to improve exclusive breastfeeding amongst mothers in a cluster randomised controlled trial in South Africa. BMC Health Serv Res. 2020;20(1):1–8.

25. Alioth Finance. South Africa Inflation Calculator [Internet]. 2024 [cited 2024 Jun 5]. Available from: https://www.officialdata.org/south-africa/inflation/2013?endYear=2023&amount=1720.34

26. Mapanga W, Norris SA, Craig A, Ayeni OA, Chen WC, Jacobson JS, et al. Drivers of Disparities in Stage at Diagnosis Among Women With Breast Cancer: South African Breast Cancers and HIV Outcomes Cohort. Prepr (Version 1) available Res Sq [Internet]. 2022; Available from: https://doi.org/10.21203/rs.3.rs-1180376/v1

27. Daviaud E, Subedar H. Staffing Norms for Primary Health Care in the context of PHC Re-engineering - Report to the National Department of Health. Dep Heal Med Res Counc [Internet]. 2012;(October):1–29. Available from: http://www.mrc.ac.za/sites/default/files/files/2016-07-14/StaffingNorms.pdf

28. Public Health and Social Development Sectoral Bargaining Council. Resolution 3 of 2022 - Agreement on standardisation of renumeration of community health workers in the department of health. [Internet]. 2022. Available from: https://www.phsdsbc.org.za/wp-content/uploads/2022/07/RESOLUTION-03-OF-2022-EXTENSION-OF-RESOLUTION-01-OF-2021-AGREEMENT-ON-STANDARDISATION-OF-RENUMERATION-OF-COMMUNITY-HEALTH-WORKERS.pdf

29. Blecher MS, Day C, Dove S, Cairns R. Primary Health Care financing in the public sector : Primary Health Care : systems support. South African Heal Rev [Internet]. 2008;179. Available from: http://0-

search.ebscohost.com.brum.beds.ac.uk/login.aspx?direct=true&db=edssas&AN=edssas.healthr.2008.a15&site=eds-live&scope=site
