# Supplementary material for: Equity impact and cost-effectiveness of a community health worker breast cancer educational programme in rural South Africa: a modelling study
Source: BMJ Open. 2026 Apr 21;16(4):e114908. doi: 10.1136/bmjopen-2025-114908 (PMC13110530; doi:10.1136/bmjopen-2025-114908)
Supplement: online supplemental file 2 [file bmjopen-16-4-s002.docx]

*Table S14: Comparison of model outputs per person at various discount rates.*

| **Discount rate** | **0%** | **3%** | **5% (base case)** | **10%** |
| --- | --- | --- | --- | --- |
| **Life-years saved** | 0.687  (0.604;0.758) | 0.448  (0.397;0.493) | 0.356  (0.316;0.391) | 0.226  (0.203;0.248) |
| **Incremental QALYs** | 0.615  (0.54;0.678) | 0.395  (0.351;0.434) | 0.310  (0.277;0.340) | 0.192  (0.173;0.210) |
| **Incremental Costs (R)** | 13,541  (13,139;13,935) | 13,522  (13,135;13,887) | 13,513  (13,135;13,861) | 13,499  (13,141;13,810) |
| **ICER** | 22,113  (19,764;25,121) | 34,344  (30,833;39,023) | 43,706  (39,340;49,567) | 70,664  (63,878;79,214) |
| **NMB 1 (ZAR)** | 22,113  (17,829;25,925) | 9,404  (6,620;11,802) | 4,487  (2,339;6,355) | -2,382  (-3,656; -1,227) |
| **NMB 2 (ZAR)** | 53,762  (45,697;60,793) | 29,736  (24,668;34,137) | 20,449  (16,570;23,857) | 7,477  (5,220;9,551) |
| **NMB 3 (ZAR)** | 188,372  (164,322;209,170) | 116,253  (101,468;129,184) | 88,376  (77,126;98,339) | 49,429  (42,990;55,426) |
