# Supplementary material for: Equity impact and cost-effectiveness of a community health worker breast cancer educational programme in rural South Africa: a modelling study
Source: BMJ Open. 2026 Apr 21;16(4):e114908. doi: 10.1136/bmjopen-2025-114908 (PMC13110530; doi:10.1136/bmjopen-2025-114908)
Supplement: Supplementary file 3 [file bmjopen-2025-114908.reviewer_comments.pdf]

PEER REVIEW HISTORY

BMJ Open publishes all reviews undertaken for accepted manuscripts. Reviewers are asked to complete a checklist review form and are provided with free text boxes to elaborate on their assessment. These free text comments are reproduced below.

ARTICLE DETAILS

Title (Provisional)

Equity Impact and Cost-Effectiveness of a Community Health Worker Breast Cancer Educational Programme in Rural South Africa: A Modelling Study.

Authors

Chanakira, Esther Zvikomborero Zvikomborero; Thomas, Chloe; Miot, Jacqui; Mandrik, Olena

---

VERSION 1 - REVIEW

**Reviewer**	**1**
**Name**	**Bhattarai, Nawaraj**
**Affiliation**	**Institute of Health & Society, Newcastle University**
**Date**	**06-Feb-2026**
**COI**	**None**

This is a beautifully and clearly written manuscript with all the methodological details provided in the main body as well as in the supplementary files. I have no major concerns and suggest that this be accepted for publication (this happens rarely, but it is the case here). It would be good if the authors address these few minor issues:

1. Could the authors provide a rationale for their assumption in page 8, line 18, where they say "These estimates were extrapolated to 10 years, after which an assumption of no further breast cancer deaths was applied."

2. Could the authors present the Table 3, as they have presented Table 2. (they may keep the current content of Table 3, but would need additional information presented as in Table 2). This would allow visualisation of the impact of parameter uncertainties in costs and outcomes.

I would like to see this published. Good luck

**Reviewer**	**2**
**Name**	**Yano, Yoshitaka**
**Affiliation**	**Kyoto Pharmaceutical University, Education and Research**

**Date**	**06-Mar-2026**
**COI**	**None**

This manuscript describes the impact of an educational program for community health worker on QOL of the breast cancer patients in rural South Africa. The analysis results suggested that the education program leads to improved survival time and cost-effectiveness through early detection and improved QOL of the patients.

The analysis was performed in detail based on a complicated model and parameter settings, but it seems difficult to fully assess the accuracy/reliability of each parameter's values, and this leads to making robustness of the results unclear for readers. Furthermore, the results obtained may not be significant if we consider inter-individual variability of the parameters among patients. While I acknowledge the effectiveness of education, we must be cautious about the clinical utility of such model analysis results that depend only on average parameters.

As is common in cost-effectiveness analyses, the analysis often uses only average values for parameters, ignoring individual variability. While it would have been possible to conduct a sensitivity analysis for all parameters used, this would have been so technical.

The numbers in parentheses in each table seem to represent 95% confidence intervals for the estimates, but the table explanations are unhelpful. The confidence intervals sometimes overlap between those in the current care arm and in the intervention arm, making it difficult to accept a significant difference between them.

I, as a reviewer, suggest simpler presentation of the results that the authors can insist on the usefulness of the education program, even if the results are in the limited area in the country, for example, simply by showing the Kaplan-Meier plots of each group with the statistical analysis.

VERSION 1 - AUTHOR RESPONSE

Reviewer 1
Thank you very much for your time and for the thoughtful and encouraging feedback on our manuscript. We greatly appreciate your positive evaluation of the work and are grateful for your constructive suggestions and have carefully addressed the issues you raised in the revised version of the manuscript.

Comment 1:
Could the authors provide a rationale for their assumption in page 8, line 18, where they say "These estimates were extrapolated to 10 years, after which an assumption of no further breast cancer deaths was applied."

Thank you for this helpful comment. We agree that the rationale for this assumption should be clarified. We have now expanded the text to provide further justification. The revised

section on page 8 line 20-24 now reads: "These estimates were extrapolated to 10 years, after which an assumption of no further breast cancer deaths was applied. This assumption is supported by clinical evidence and epidemiological data, including long-term survival patterns reported by the Surveillance, Epidemiology, and End Results (SEER) programme in the USA, which demonstrates that breast cancer mortality risk significantly declines several years after diagnosis and approaches that of the general population beyond the 10-year mark (18)."
National Cancer Institution. SEER website. 2019. SEER Cancer Statistics Review. 1975-2016.

Comment 2:
Could the authors present the Table 3, as they have presented Table 2. (they may keep the current content of Table 3 but would need additional information presented as in Table 2). This would allow visualisation of the impact of parameter uncertainties in costs and outcomes.

We have revised the table to include the Net Monetary Benefit (NMB) values for each model arm, in addition to the incremental values that were already presented, consistent with the format used in Table 2.

Reviewer 2
We sincerely thank the reviewer for the careful reading of our manuscript and for providing thoughtful and constructive comments. We appreciate the time and effort invested in evaluating our work. The suggestions have been very helpful in improving the clarity, rigor, and overall presentation of the manuscript.

Comment 1
The analysis was performed in detail based on a complicated model and parameter settings, but it seems difficult to fully assess the accuracy/reliability of each parameter's values, and this leads to making robustness of the results unclear for readers.

We thank the reviewer for this important comment regarding the complexity of the model and the reliability of the parameter values used. We acknowledge that the model incorporates multiple parameters and assumptions, which may make it challenging for readers to fully assess the robustness of each parameter individually.
Within the manuscript, we provide a summary of the modelling approach and key parameter values in the main text to enhance clarity and accessibility. To further improve transparency, we have now explicitly signposted the availability of detailed parameter information in the supplementary materials within the main text (page 8, lines 13–14): "Full model parameter details, including sources, assumptions, and uncertainty ranges, are provided in the supplementary methods, with a summary presented here." The supplementary materials include comprehensive information on parameter sources, assumptions applied where empirical data was limited, and the rationale underpinning parameter selection. This structure is intended to balance readability of the main manuscript with full methodological transparency, enabling readers to explore the underlying detail as needed.
To further address parameter uncertainty, we conducted probabilistic sensitivity analyses

using appropriate probability distributions assigned to the model parameters. Details on the specific distributions used are provided in Table S14 in supplementary methods. This approach allowed us to evaluate the impact of parameter uncertainty on the model outcomes. Additionally, where greater uncertainty was identified for specific parameters, we performed scenario analyses using alternative plausible parameter values. The rationale for these scenarios and the associated assumptions is described in detail in the supplementary methods. We also acknowledge the data-related limitations associated with generating some of the parameters within the model. These limitations are discussed in the manuscript (Discussion, page 23), including the reliance on intervention effectiveness estimates from a Rwandan study, the adaptation of cost data from a breastfeeding programme, the extrapolation of mortality estimates beyond the available three-year ABC-DO dataset, and the use of HRQoL values derived from a study conducted in China. These factors introduce potential contextual and data-related uncertainties. However, we addressed these limitations by incorporating parameter uncertainty into the model and by conducting additional sensitivity and scenario analyses to explore their potential impact on the results. We also highlight that future studies using South Africa specific data would further strengthen the evidence base.

Comment 2
Furthermore, the results obtained may not be significant if we consider inter-individual variability of the parameters among patients. While I acknowledge the effectiveness of education, we must be cautious about the clinical utility of such model analysis results that depend only on average parameters. As is common in cost-effectiveness analyses, the analysis often uses only average values for parameters, ignoring individual variability.

We thank the reviewer for this important comment regarding the potential impact of inter-individual variability on the model results. We agree that patient heterogeneity can influence outcomes and should be considered where possible.
With respect to the intervention effect, we acknowledge that ideally this would vary across population subgroups. It is plausible, for example, that the effectiveness of educational interventions differs by education level or socioeconomic position. However, we did not have sufficient empirical data to parameterise subgroup-specific intervention effects. The available evidence utilised in the study did not report heterogeneity in effectiveness across relevant subgroups. As a result, the intervention effect was applied uniformly across the rural population, which represents a limitation of the current analysis. This limitation has now been explicitly acknowledged in the limitations paragraph of the discussion section in the manuscript (page 22, lines 12–17): “The intervention effect was derived from a Rwandan study, which may limit generalisability to the South African setting. In addition, the intervention effect was applied uniformly across the model population. It is plausible that the effectiveness of educational interventions may vary across population subgroups, for example according to education level or socioeconomic position. However, insufficient data was available to parameterise subgroup-specific intervention effects.”
Inter-individual variability in mortality outcomes was incorporated where appropriate data was available. The ABC-DO study used to inform all-cause mortality reported statistically and clinically significant differences by age, stage at diagnosis, race, place of residence, socioeconomic position, and level of education. Based on these data, we incorporated heterogeneity into the model by allowing mortality rates to vary according to stage at

diagnosis, place of residence, and highest level of education. The methods used to determine which characteristics were incorporated into the model are described in the supplementary methods. As noted in the Methods section of the main manuscript (page 8, line 18-19), these factors were selected as the most appropriate variables for representing patient-level variability within the modelling framework. Supplementary methods include graphical illustrations of differences in the probability of death across these characteristics obtained through model calibration, alongside the hazard ratios derived from the ABC-DO study.

Comment 3
While it would have been possible to conduct a sensitivity analysis for all parameters used, this would have been so technical.

We thank the reviewer for this important comment regarding the assessment of parameter uncertainty and apologise that the description of this aspect of the analysis was not sufficiently clear in the original manuscript. We have now revised the Cost-effectiveness Analysis section under Methods to improve clarity around how parameter uncertainty is handled within the model (page 11, lines 13-23).
In this analysis, we conducted probabilistic sensitivity analysis (PSA), in which probability distributions are assigned to model parameters and propagated through the model simultaneously. This approach captures the joint uncertainty across all parameters and ensures that results are not based solely on point estimates but instead reflect the combined impact of parameter uncertainty on model outcomes. The specific distributions and parameter ranges used are reported in Table S10 in the supplementary methods, including the associated 95% confidence intervals of the parameters. PSA was conducted for all model parameters except for the age-standardised mortality rate (ASMR), which was derived from national-level data sources which have relatively limited uncertainty.
In addition to PSA, we conducted scenario analyses for selected parameters where uncertainty may not be fully captured by probability distributions alone, or where structural assumptions were required. These included intervention costs and effects, mortality rates, and assumptions used for extrapolating mortality beyond the available data period. The rationale and parameter values for these scenarios are described in detail in the supplementary methods.
York Health Economics Consortium. YHEC website. 2016. Probabilistic / Stochastic Sensitivity Analysis. https://www.yhec.co.uk/glossary-term/probabilistic-stochastic-sensitivity-analysis/

Comment 4

The numbers in parentheses in each table seem to represent 95% confidence intervals for the estimates, but the table explanations are unhelpful. The confidence intervals sometimes overlap between those in the current care arm and in the intervention arm, making it difficult to accept a significant difference between them.

Thank you for this important comment. We apologise for the lack of clarity in the original manuscript.
The values shown in parentheses represent 95% credible intervals derived from the

probabilistic sensitivity analysis (PSA), rather than confidence intervals. Credible intervals arise from a Bayesian framework and represent the range within which 95% of the simulated values fall, given the joint uncertainty in the model parameters. In contrast, confidence intervals are derived from a frequentist framework and reflect the range that would contain the true parameter value across repeated samples, rather than the distribution of outcomes from a single model. This distinction is particularly relevant in decision-analytic modelling, where PSA is used to propagate parameter uncertainty through the model and characterise uncertainty in outcomes.

With respect to the overlap between intervals in the current care and intervention arms, credible intervals describe the uncertainty around each strategy's estimate individually. Overlap between these intervals does not necessarily imply the absence of meaningful differences between strategies. Instead, comparisons are evaluated using incremental outcomes calculated within each PSA simulation, which capture the joint uncertainty across parameters. Decision-relevant results are therefore based on these incremental measures (e.g., incremental costs, QALYs, ICERs, and net monetary benefit).

To clarify this, we have added the following to the Methods section (page 11, lines 15-23): "Results from the PSA were presented as mean estimates with corresponding 95% credible intervals, representing the 2.5th and 97.5th percentiles of the simulated outcome distributions. These intervals reflect parameter uncertainty around the estimated outcomes for each strategy. Comparative assessments between strategies were based on incremental outcomes and cost-effectiveness measures derived from the probabilistic simulations." We have now also specified in our table headings that the results presented are the mean value and the associated 95% credible interval.

Hespanhol L, Vallio CS, Costa LM, Saragiotto BT. PMC website. 2019. Understanding and interpreting confidence and credible intervals around effect estimates.

Comment 5

I, as a reviewer, suggest simpler presentation of the results that the authors can insist on the usefulness of the education program, even if the results are in the limited area in the country, for example, simply by showing the Kaplan-Meier plots of each group with the statistical analysis.

We thank the reviewer for the suggestion regarding a simpler presentation of the results. We have improved the figure and table headings to improve interpretability of results:

Page 15 - "Table 3: Cost-effectiveness results across WTP thresholds, reported as mean with 95% CrI."

Page 15 - "Figure 2: Cost-effectiveness Acceptability Curve (CEAC) showing the probability that the intervention is cost-effective across different willingness-to-pay (WTP) thresholds."

While Kaplan–Meier curves are commonly used to present survival data, they would not fully capture the complexity and interactive effects included in our model. Specifically, our analysis incorporates individual-level heterogeneity in mortality, stage at diagnosis, place of residence, and education level, as well as the impact of the intervention on stage shifting and mortality. Kaplan–Meier curves would therefore oversimplify these effects and could obscure important interactions that are captured in the current model outputs.

For the inequality analysis, we present differences in age-standardised mortality rates (ASMR) over a 10-year period to enable the equity impact to be assessed across racial and

residence populations while nullifying the impact of the age structures of the groups. Kaplan Meier curves would not enable this to be captured.

Similarly, the cost-effectiveness results follow standard presentation formats used in health economic evaluations and are designed to allow comparison across model arms, including uncertainty intervals.
